# A Systematic Review on Amnion as a Cell Delivery Scaffolding Material for Cartilage Regeneration in Pre-Clinical and Clinical Studies

**DOI:** 10.3390/bioengineering13030357

**Published:** 2026-03-18

**Authors:** Shu-Yong Liow, Sik-Loo Tan, Alvin Jiunn-Hieng Lu, Kwong Weng Loh, Seow Hui Teo, Chan Young Lee, Le Wan, Azlina Amir Abbas, Kyung-Soon Park

**Affiliations:** 1 National Orthopaedic Center of Excellence for Research & Learning (NOCERAL), Department of Orthopaedic Surgery, Faculty of Medicine, Universiti Malaya, Kuala Lumpur 50603, Malaysia; liowshuyong@gmail.com (S.-Y.L.); alu57508@gmail.com (A.J.-H.L.); melvinloh@um.edu.my (K.W.L.); tseowhui@um.edu.my (S.H.T.); 2 Department of Orthopedic Surgery, Chonnam National University Medical School and Hwasun Hospital, Hwasun-gun 58128, Jeollanam-do, Republic of Korea; cnuhoslee@gmail.com (C.Y.L.); wle202302@gmail.com (L.W.)

**Keywords:** mesenchymal stem cells, amnion, orthopaedics, cartilage, tissue engineering, musculoskeletal sciences, regenerative medicine, full-thickness cartilage defects, scaffold, biomaterials

## Abstract

Cartilage is an important yet vulnerable tissue with limited self-healing capacity, where damage often progresses to joint degeneration, which eventually leads to severe osteoarthritis (OA). Current tissue engineering strategies focus on biocompatible scaffolds for cartilage regeneration, particularly amnion (or amniotic membrane), emerging as a promising biomaterial due to its wide availability, low immunogenicity, and naturally derived microenvironment that is advantageous for cartilage regeneration. This systematic review aims to evaluate the existing evidence on the efficacy of amnion as a tissue scaffolding material for cartilage regeneration in both preclinical and clinical studies. Using terms such as “cartilage damage”, “cartilage injuries”, “amnion” and “amniotic membrane”, 19 relevant studies were identified across three major databases (PubMed, Scopus and Web of Science) until 25 December 2025. All preclinical and clinical studies that utilized amnion for cartilage repair or as cartilage tissue engineering scaffolding materials were included. Evidence quality was assessed using the OHAT and MINORS risk of bias tool. This study is prospectively registered in the PROSPERO database under the ID 1178444. The findings consistently indicate that amniotic scaffolds, regardless of processing methods or cell seeding, yield favorable outcomes without adverse effects across different species. In vitro analysis revealed that treatment groups with amnion show better cell attachment, viability, and proliferation, and higher content of cartilage-related markers expressed by the seeded cells, either chondrocyte, bone marrow-derived mesenchymal stem cells (MSCs), adipose tissue-derived MSCs, placenta-derived MSCs, umbilical cord-derived MSCs, amniotic MSCs or amniotic epithelial cells. In in vivo and ex vivo studies, amnion-treated groups demonstrated improved quality of the treated cartilage, with better integration, as indicated by higher histological scores and the presence of type II collagen (COL-II). There was an inconsistency in the reporting of cartilage defect dimensions in the in vivo models across the different studies. Nevertheless, the outcome measurements were consistently reported with histological analysis, with or without International Cartilage Repair Society (ICRS) scoring and immunohistochemistry (IHC) analysis, across the studies. Clinically, most subjects show improvement in the Knee Injury and Osteoarthritis Outcome Score (KOOS) Sports and Recreation score and KOOS Quality of Life score, as well as reduced Visual Analogue Scale (VAS) average and maximum pain scores. In conclusion, preclinical and clinical studies support amnion as an ideal scaffold material for cartilage tissue engineering and regeneration. Future research should focus on optimizing and standardizing amnion scaffold preparation at a production scale to facilitate the translation of these positive outcomes into clinical applications. This study is funded by the Ministry of Higher Education Malaysia via Prototype Research Grant Scheme (PRGS/1/2021/SKK01/UM/02/1) and UM International Collaboration Grant—2023 SATU Joint Research Scheme Program: ST007-2024.

## 1. Introduction

Cartilage is a type of resilient connective tissue that plays an important role in facilitating flexible movement by functioning as a cushion and lubricant between the joints. While cartilage injury may not be immediately life-threatening, it can significantly reduce quality of life by causing pain, stiffness and functional deficits [1]. Focal cartilage injuries are commonly caused by acute traumatic impacts or repeated microinjuries over time and can occur at any age. These injuries can extend to different layers of the joint, including partial- and full-thickness cartilage injuries that involve only the cartilage layer, as well as osteochondral injuries that also involve the underlying subchondral bone [2,3]. The avascular and aneural nature of cartilage and low cell chondrocyte density limit its self-healing capacity. Any form of cartilage injury can predispose individuals to progressive joint degeneration and ultimately lead to osteoarthritis (OA), a more severe condition that affects the entire joint [4,5].

Many approaches have been established to treat cartilage injuries. These include palliative treatments that primarily aim to alleviate symptoms, as well as surgical techniques designed to achieve cartilage repair through bone marrow stimulation or regenerative modalities. Bone marrow stimulation techniques, such as microfracture, allow the infiltration of bone marrow stem cells into the injured site but typically result in fibrocartilage formation, which is less durable than native hyaline cartilage. Meanwhile, existing regenerative modalities, such as osteochondral graft transplantation and autologous chondrocyte implantation, also encounter issues, including limited graft availability and the risk of graft failure [6,7,8]. These limitations have highlighted the need to introduce more advanced tissue engineering strategies for effective cartilage regeneration. Tissue engineering is a complex field that utilizes a combination of cells, scaffold materials, and growth factors to repair and restore the structure and functionality of tissues [9,10,11]. Scaffolds derived from natural or synthetic biomaterials provide a supportive framework for cell attachment and development and facilitate the integration of newly generated tissue with the host [12,13].

Since the amnion (or amniotic membrane) was first introduced for skin transplantation in 1910, its use has significantly expanded to various medical fields, including orthopedics. This growth is largely attributed to its unique biological and mechanical properties [14]. In terms of biological properties, amnion exhibits immunosuppressive, antifibrotic, antimicrobial and anti-inflammatory effects, which are desirable features for transplantation [15,16]. Regarding biomechanical properties, amnion possesses flexibility, rigidity, and tensile strength. Together with its extracellular matrix (ECM), which mimics the native environment for cartilage regeneration, these features make the amnion an ideal scaffold, offering high immune tolerance, minimal complications, and effective cartilage tissue regeneration [17,18].

Despite these promising characteristics, the application of amnion as a tissue engineering scaffold is comparatively well established in other fields such as wound healing and ophthalmology. Although there is growing interest in the amnion for cartilage regeneration, there are currently no comprehensive systematic reviews that consolidate reported outcomes across all preclinical (in vitro, in vivo, and ex vivo) and clinical studies covering the broad spectrum of cartilage injuries. Therefore, this systematic review aims to evaluate the existing evidence on the efficacy of amnion as a tissue scaffolding material for cartilage regeneration in both preclinical and clinical studies, to guide future research directions and facilitate clinical translation. In this review, the preclinical studies, both in vitro and ex vivo, showed evidence of the amnion as a biomaterial that supports chondrocyte proliferation and sustains chondrocyte phenotypic expression and chondrogenic differentiation in multipotent mesenchymal stem cells (MSCs).

In the in vivo preclinical models, both leporine (rabbits) and ovine (sheep) models showed hyaline cartilage-like tissue formation at the repaired site post-treatment with amnion-derived biomaterials, either with or without cells seeded onto the amnion. One clinical study used the commercially available hypothermically stored amnion (HSAM) to treat chondral lesions with International Cartilage Repair Society (ICRS) grade 3 or grade 4A (of the femur). It reported improved KOOS Sports and Recreation and Quality of Life (QoL) scores, in addition to improvements in VAS Average Pain and Maximum Pain scores, at 24 months compared to baseline. The Modified Magnetic Resonance Observation of Cartilage Repair Tissue (MOCART) scoring based on MRI scans showed that 7 of 10 subjects had complete defect repair and filling by 24 months. Three out of the 10 enrolled subjects reported at least one mild to moderate adverse event, though none were related to the use of amnion. This systematic review summarized the current evidence supporting the efficacy of amnion as a tissue scaffolding material for cartilage regeneration.

## 2. Materials and Methods

This systematic review was conducted in accordance with the Preferred Reporting Items for Systematic Reviews and Meta-Analyses (PRISMA) guidelines [19]. The study protocol was prospectively registered in the PROSPERO database under the ID 1178444. The complete PRISMA 2020 checklist has been provided to the editorial office as part of the submission documentation.

### 2.1. Search Strategy

We conducted a systematic search for research articles published over the last 25 years (from January 2000 to December 2025; up to 25 December 2025) across three primary databases: PubMed, Web of Science, and Scopus. The search was conducted using selected keywords (i.e., “cartilage damage”, “cartilage injuries”, “amnion” and “amniotic membrane”), combined with Boolean operators to identify articles with relevant titles, abstracts or keywords. The specific search strategies applied for each database are shown in Table A1. All non-redundant search outputs from all the databases (with their titles and abstracts) were uploaded to the Abstrackr for the screening process. All records were independently screened by two reviewers (S.-Y.L. and A.J.-H.L.) using Abstrackr, followed by full-text assessment. Conflicts that arose among the two reviewers were addressed through team discussion and full-text assessment until a consensus was reached. The articles included from the Abstrackr screening process were also re-evaluated together in a team discussion to ensure the finalized list of the included articles met all the inclusion/exclusion criteria. Subsequently, the reference lists of the included articles were manually screened to identify any additional relevant studies.

### 2.2. Selection (Eligibility) Criteria

All preclinical (in vitro, ex vivo, and in vivo) and clinical studies on the application of any form of amnion as scaffolds for cartilage regeneration were included. This encompasses the use of amnion alone, amnion–chorion composites, or amnion combined with other biomaterials, while studies utilizing the chorion layer alone were excluded. Studies involving only the utilization of cells derived from amnion were also excluded. Only research articles published in English were selected. The detailed inclusion and exclusion criteria were listed in Table 1.

This current review focused on focal cartilage defects, and OA is regarded as an exclusion criterion. It is important to differentiate between these two conditions, as both involve cartilage injury but differ in pathology and relevance to the review. Focal traumatic defects typically occur locally within a relatively healthy joint, allowing for a controlled assessment of scaffold-based treatment. On the other hand, OA is a systemic joint disease characterized by chronic pro-inflammatory conditions with elevated catabolic cytokines that affect the whole joint. This environment might impair healing and diminish the effects of the amnion on tissue regeneration [20]. Thus, the exclusion of OA models is essential to avoid the confounding effects on the scaffold performance and ensure the validity of our findings regarding amnion’s efficacy for localized cartilage regeneration.

### 2.3. Data Collection

To harmonize the terminology used in different articles, the term “amnion” is used to collectively describe the “amnion scaffold”, “amniotic membrane”, “acellular amniotic membrane”, and “HAM” reported in the different studies. The term ‘amnion” covers multiple preservation/processing methods, where the preservation/processing methods will be added as a prefix to differentiate the “amnion” preserved/processed by different methods, e.g., “glycerol-preserved amnion” was used to describe the “amnion” preserved in glycerol; “air-dried amnion” was used to describe amnion processed by the air-drying method. The term “fresh amnion” is used to describe any “amnion” used in the studies without any preservation. The term “intact” is used to describe “amnion” without decellularization/de-epithelialization processing.

Data were extracted from the included articles using a standardized Excel spreadsheet by the first reviewer and checked by the second reviewer to ensure accuracy. General data extraction focused on: the details of the articles (e.g., author, article title, journal, year of publication and aim of study), amnion sources, procurement criteria and amnion preparation methods; types of cell/composites applied on the amnion; and animal species, defect models and dimensions. For in vitro studies, the findings for the chondrogenic marker expression profiles were tabulated in the data charting spreadsheet. For ex vivo and in vivo *studies*, data extracted to the spreadsheets include the gross observations (with and without ICRS scores), histological examinations (with and without scoring) and immunohistochemical (IHC) staining for hyaline cartilage-related markers. In addition, patient-reported outcome measures such as the Knee Injury and Osteoarthritis Outcome Score (KOOS) and the Visual Analogue Scale (VAS) scores were extracted from the included clinical study. Studies lacking reported results for a specific section in the spreadsheets were marked as “Not Reported” or “NR”.

Meta-analysis was not performed due to the considerable heterogeneity observed in the study methodologies, including variations in amnion preparation techniques, the animal models employed, the types of defects created, and the cell types seeded. Furthermore, standardized quantitative outcome measures were lacking for synthesizing the data for further statistical comparisons. Therefore, qualitative synthesis of the evidence was conducted in this systematic review.

## 3. Results

### 3.1. Selection of Sources of Evidence

A total of 110 records were retrieved from all databases. During the initial screening stage, prior to title and abstract screening, all non-English papers, review articles, and duplicates were removed manually (using EndNote Citation Manager). Of the 90 articles excluded at this stage, half were related to OA. A total of 20 articles proceeded to the title and abstract screening in the Abstrackr. The reviewers reached an initial agreement of 85% (17/20; Table A2). After conflicts were resolved, only ten articles (out of the 20 articles) met all the inclusion/exclusion criteria and proceeded for full-text review. Most excluded articles were articles reporting on the use of amnion-derived stem cells and amniotic fluid rather than the amnion itself.

**Figure 1:**
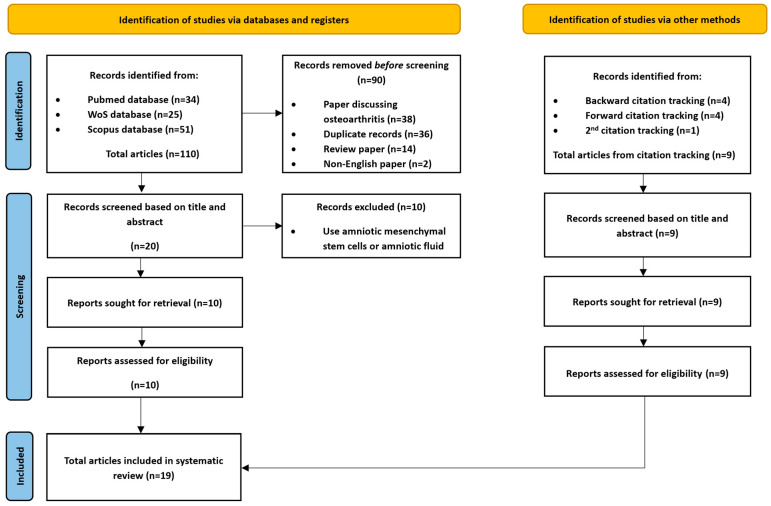
PRISMA workflow for the overall process of article selection in this systematic review. In total, 19 articles were selected for this systematic review, based on the stated inclusion and exclusion criteria.

Subsequently, manual citation tracking was performed to identify any additional relevant records that might have been missed during the keyword searches. A total of nine additional studies were included from citation tracking. Ultimately, 19 articles were included in this systematic review (Figure 1). All these 19 articles reported on using amnion alone, and none of these studies used amnion/chorion in their studies.

### 3.2. Characteristics of Source of Evidence

This systematic review included a total of 19 articles published between 2007 and 2024. During the screening process, 10 studies were excluded for the following reasons: utilization of amnion-derived mesenchymal stem cells (AMSCs) on scaffolds without amnion (n = 4); use of AMSCs as the scaffolding materials instead of amnion (n = 3); use of amniotic fluid (n = 1); application of whole decellularized placenta rather than the isolated amnion membrane (n = 1); and the application of amnion membrane extract in culture medium instead of acting as a scaffold (n = 1). All excluded studies are listed in Table A3. Two articles reporting on cartilage ex vivo models [21,22] were reviewed, with a conflict between the two reviewers as the studies used osteoarthritic cartilage samples. The final decision made after discussion was to include these two articles because both aimed to evaluate the amnion as a scaffold for cartilage repair, utilizing osteoarthritic cartilage tissue samples as an ex vivo cartilage injury model rather than as an ex vivo OA model. The details of all 19 included articles are summarized in Table 2. 

The risk of bias for the included studies was assessed using the Office of Health and Translation (OHAT) risk of bias tool for preclinical studies and the Methodological Index for Non-Randomized Studies (MINORS) tool for the clinical study. Detailed individual assessments are provided in Table A4 (OHAT) and Table A5 (MINORS).

**Figure 2:**
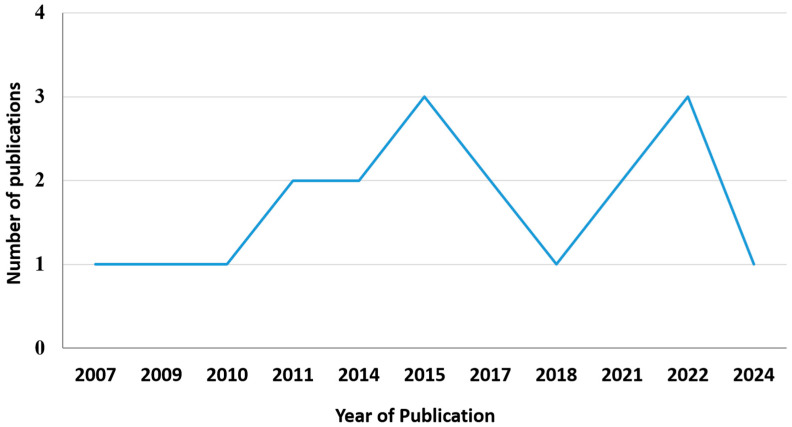
The number of studies included in the systematic review from 2007 to 2024.

**Figure 3:**
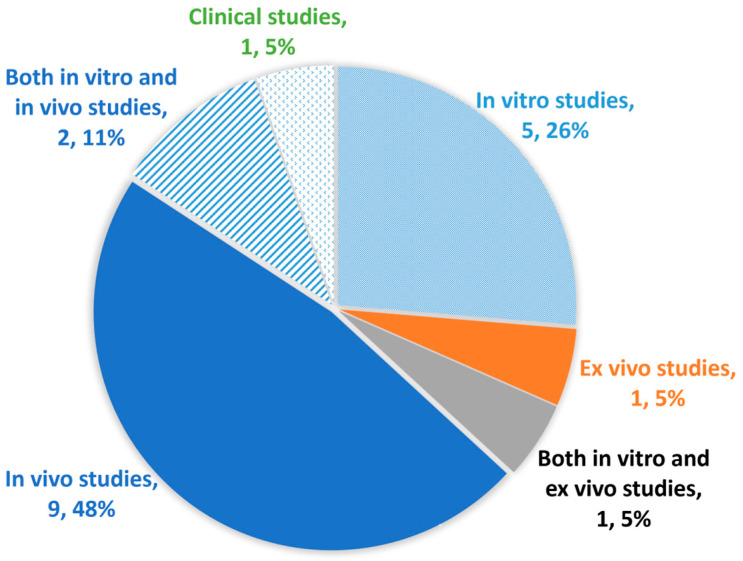
Pie chart showing the distribution of study types included in this systematic review (n=19). A total of five (26%) were in vitro studies, one (5%) was ex vivo study, nine (48%) were in vivo studies, and only one (5%) was a clinical study. Besides, one (5%) study combined both in vitro and ex vivo models, while two (11%) studies combined both in vitro and in vivo experiments.

**Figure 4:**
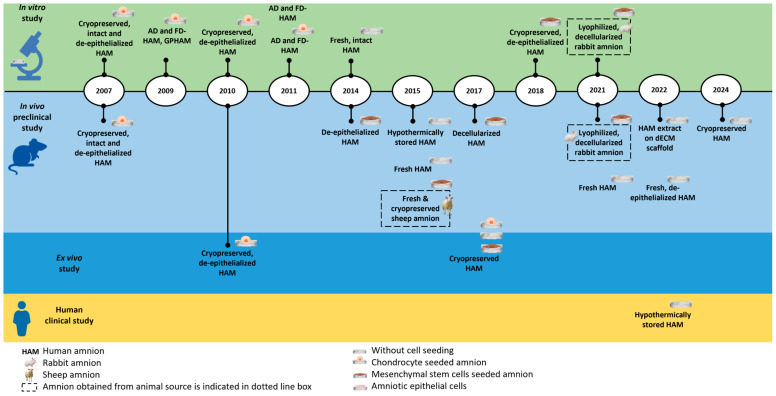
Timeline of publications on amnion and cartilage included in this systematic review (n = 19). The initial applications of amnion in cartilage-related research used intact and denuded (de-epithelialized) amnion. From 2009 onward, studies also reported other processing methods, including air-drying, freeze-drying (lyophilization), cryopreservation, and hypothermic storage. Acronym: AD HAM: air-dried human amnion; FD HAM: freeze-dried human amnion; GPHAM: glycerol-preserved human amnion; Fresh: amnion without any preservation step; Intact: amnion without any de-epithelization/decellularization step.

Overall, the total number of publications on the use of amnion as a scaffold for cartilage regeneration has remained relatively low but constant over the years, ranging from one to three publications per year (Figure 2). This indicates sustained interest in this research field. Among these studies, five were in vitro, one was ex vivo, and nine were in vivo (Figure 3). Additionally, one study combined both in vitro and ex vivo models, while two studies combined both in vitro and in vivo experiments. Only one clinical study was identified in this review. This highlights that current research is predominantly at the preclinical level, underscoring the need for further clinical investigations to establish its therapeutic feasibility. The summary for the publication timeline of all 19 articles is shown in Figure 4.

### 3.3. Source and Processing Techniques of Amnion

The systematic review included studies that utilized human amnion (HAM) as the primary source for cartilage regeneration scaffolds, with 89% (17/19) of the studies employing human-derived amnion. The amnion was typically obtained from healthy women undergoing caesarean sections and screened for infectious diseases, particularly blood-borne diseases, including human immunodeficiency virus (HIV), Hepatitis B virus (HBV), Hepatitis C virus (HCV), and syphilis (Table 3). In addition to human-derived amnion, one study used amnion sourced from rabbits [29] and another from sheep [31].

**Figure 5:**
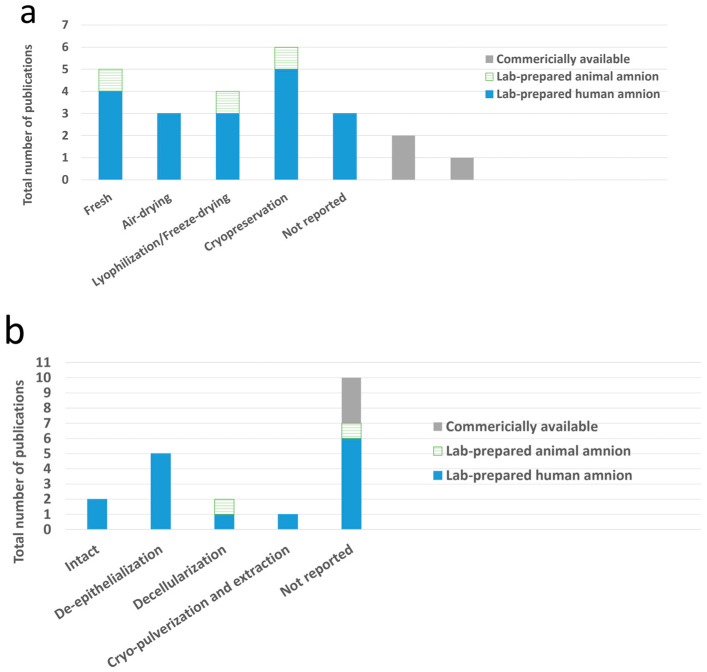
The summary of different preservation methods and processing used in the selected studies (n = 19). The stacked bar chart showing the frequency of various preservation (**a**) and processing methods (**b**) indicated a high frequency of studies using the cryopreservation technique and de-epithelialization processing.

Preservation methods for the amnion varied among the selected studies (Figure 5a), encompassing fresh, air-dried, lyophilized (freeze-dried), and cryopreserved amnions. Five studies utilized fresh amnion, six used cryopreserved, three used air-dried and four used freeze-dried. Notably, some studies employed commercially available products, including glycerol-preserved human amnion (GPHAM) [23] and hypothermically stored amnion (HSAM) [33,39]; however, three studies did not specify a preservation method. Regarding processing methods (Figure 5b), four major techniques were identified: utilization of intact amnion, de-epithelialization, decellularization and cryo-pulverization, followed by extraction. The most frequently used technique was de-epithelialization (n = 5), followed by decellularization (n = 2), and used in its intact state (n = 2). One study utilized amnion extract obtained via cryo-pulverization, incorporated with a decellularized osteochondral ECM scaffold [36]. Ten studies did not specify the processing methods.

**Figure 6:**
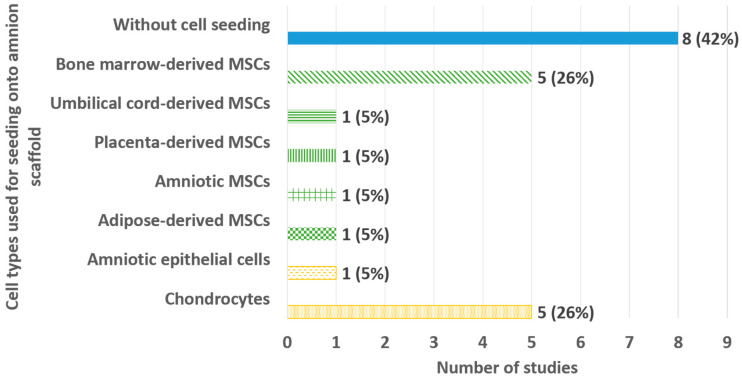
Bar chart for the summary of studies that seeded cells onto the amnion scaffold.

Cell seeding strategies were employed in 11 out of 19 studies (58%) to promote chondrogenic differentiation and tissue regenerative potential of the amnion scaffolds (Figure 6). The most commonly seeded cells were bone marrow-derived mesenchymal stem cells (BMSCs) and chondrocytes (n = 5 for each, Figure 6). Other cell types utilized in the studies included umbilical cord-derived mesenchymal stem cells (UMSCs), placenta-derived mesenchymal stem cells (PMSCs), amnion-derived stem cells (AMSCs), adipose-derived mesenchymal stem cells (ADSCs) and amniotic epithelial cells (AECs) (n = 1 for each).

### 3.4. Effects of Amnion Scaffolds in Enhancing Chondrocytes and Chondrogenic MSCs Proliferation and Phenotypic Expressions In Vitro

The articles reported on in vitro studies on amnion are summarized in Table 4. Most of the preclinical in vitro studies showed that amnion scaffolds supported cell attachment, proliferation, and chondrogenic differentiation of various cell types, including chondrocytes, bone marrow-derived mesenchymal stem cells (BMSCs), PMSCs, UMSCs and ADSCs.

Among all in vitro studies, there is only one study that did not apply cell seeding. Lindenmair et al. [26] examined the chondrogenic potential of the amnion with its native cells in different media; they found that amnion in chondrogenic media (with or without FGF) consistently showed positive effects on GAG/viability, type II collagen (COL-II) content, and cartilage-related gene expression. Boo et al. [23] found that chondrocytes proliferated on all amnion scaffold types; however, commercially available glycerol-preserved human amnion showed higher cell attachment stability compared to air-dried or freeze-dried forms. Krishnamurithy et al. [24] reported overall superior effects of amnion over monolayer culture, noting that the cell proliferation and GAG content in both air-dried (AD HAM) and freeze-dried (FD HAM) membranes were significantly higher than in monolayer culture.

Tan et al. [25] reported cell attachment on both AD and FD HAM and statistically significant increases in GAG expression in rabbit BMSCs seeded on amnion scaffolds versus monolayer controls. Naseer et al. [27] demonstrated that treated cells seeded on both HAM and plastic surfaces yielded better results compared to control cells in terms of proteoglycan and aggrecan contents and COL-II expression. Díaz-Prado et al. [22] found that the basement membrane of the amnion was a superior surface for chondrocyte seeding and differentiation compared to the epithelial side, indicated by better growth of chondrocytes and the presence of COL-II on the basement layer.

Jin et al. [28] observed high chondrocyte attachment and viability across multiple human amnion substrates, with immunohistochemistry detecting COL-II primarily in the group where the chondrocyte was seeded on the de-epithelialized stromal side. Finally, Cao et al. [29] demonstrated that the combination of an amnion scaffold, platelet-rich plasma (PRP) and ADSCs resulted in the highest levels of cell proliferation, viability, chondrogenic gene and protein expression, and COL-II production.

### 3.5. Application of Amnion Scaffolds in Ex Vivo Model

Two ex vivo studies (Table 5) were included in this review [21,22]. Both studies collected cartilage biopsies from healthy donors and patients with OA, which were sectioned into 6 mm diameter cartilage discs to examine the effects of amnion on cartilage repair. Notably, there were distinct differences in the model preparation between these two studies. While Muinos-Lopez et al. [21] created 2 mm diameter defects in the cartilage prior to amnion treatment, Díaz-Prado et al. [22] applied the amnion scaffolds directly onto a 6 mm cartilage disc without any defect creation.

Muinos-Lopez et al. [21] reported that HAM seeded with various cell types (including chondrocytes, hBMSCs, hACEs, and hAMSCs) showed no significant differences in the ICRS histological scores across the groups. Nevertheless, based on the Masson Trichrome-stained histopathology analysis, chondrocyte-seeded HAM-treated cartilage disc showed enhanced tissue integration compared to the other groups; the hAMSCs-seeded HAM-treated group exhibited a high abundance of COL-II expression in the IHC analysis. In the ex vivo study without defect creation on the cartilage discs, Díaz-Prado et al. [22] reported that only the chondrocyte-seeded HAM group observed new tissue formation on the cartilage disc compared to the HAM-only control-cartilage disc group; the new cartilage tissue formation was characterized by the presence of COL-II and the absence of type I collagen (COL-I) in IHC analysis, as well as mild proteoglycan expression, as indicated by Safranin-O (SO)-stained histopathology analysis.

### 3.6. In Vivo Application of Amnion Scaffolds in Different Animal Models and Cartilage Defect Characteristics

Among the 11 articles reporting on the in vivo cartilage defect models, eight studies (73%) used rabbit models, while the other three (27%) used sheep models. Scaffold retention issues were reported in two studies utilizing sheep models [32,33], in which both studies reported that 50% of the sheep failed to retain the amnion scaffold due to a lack of immobilization.

Defect models were generally classified by depth into three types (Table 5): partial-thickness defects (n = 1, 9%), full-thickness defects (n = 3, 27%), and osteochondral defects (n = 3, 27%); however, defect types were not specified in four studies (n = 4, 36%). Defects were predominantly created in the femoral groove, also known as the patella groove or trochlear groove (n = 5, 45%), followed by the lateral femoral condyle (n = 2, 18%) and the medial femoral condyle (n = 2, 18%, Table 5). One study [37] examined laryngeal cartilage defects at the thyroid lamina, while another study did not specify the defect location (Table 5).

There was an apparent inconsistency in the reporting of defect dimensions in the in vivo preclinical models (Table 6). For instance, some studies reported diameter only (n = 2, [28,37]), depth only (n = 1, [38]), width × length (n = 2, [31,34]), or diameter × depth (n = 3, [30,35,36]), with the dimension ranging from 3 to 7 mm (Table 6). In addition, in some studies, the cartilage defects were reported by area [33]. One study each from the rabbit and sheep models did not report the defect dimensions.

### 3.7. Assessment of Cartilage Regeneration Outcomes in In Vivo Studies Using Amnion

**Figure 7:**
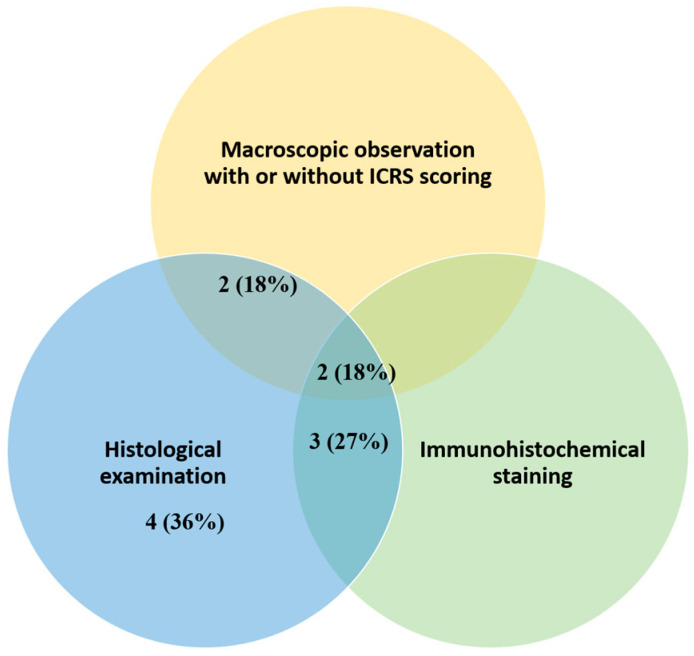
Summary of different types of evaluation used to assess the efficacy of the use of amnion for cartilage repair in in vivo studies (n = 11).

Among the in vivo studies reported in the selected articles, three main evaluation methods were used to assess the efficacy of amnion for cartilage repair in (Figure 7). All reported studies utilized histological analysis to evaluate the new cartilage formed at the study endpoint.

**Figure 8:**
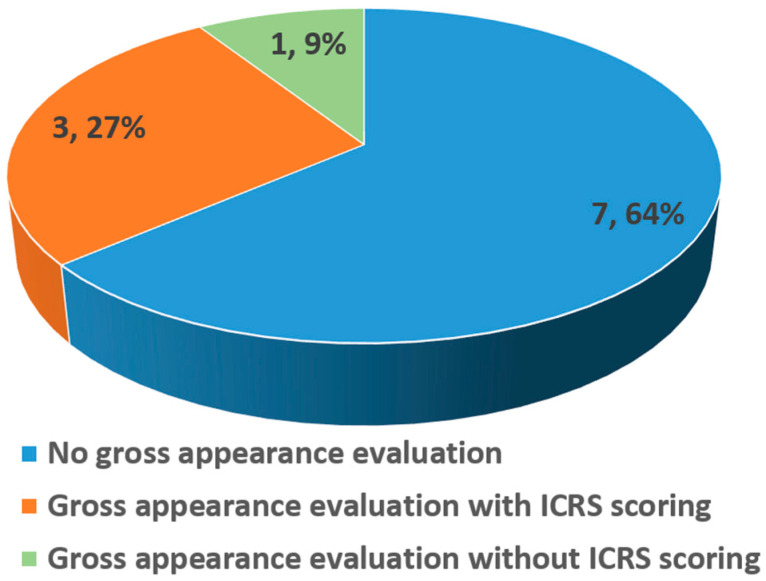
Macroscopic gross examination reported by the in vivo studies (n = 11), with or without ICRS scoring.

Macroscopic evaluation was reported in four of 11 in vivo studies (Figure 8, Table 7), with three studies reporting gross results guided by the ICRS scoring system and one reporting gross examination without a scoring system. Typically, ICRS macroscopic assessment covers the area and morphology of the newly formed tissue, as well as its integration with the surrounding native tissues [40]. An increase in the ICRS macroscopic score indicates enhanced outcome of cartilage repair [41]. All studies reporting ICRS macroscopic evaluation demonstrated superior results in the amnion-treated groups [31,35,36]. Studies by Jun et al. [35] and Rastegar Adib et al. [36] reported that the amnion-treated groups (HAAM + JCFs and dECM + amnion extract (AME), respectively) achieved the highest ICRS scores, indicating the best apparent repair, compared to the control group and other treatment groups. This is concordant with the findings where the defect area was completely covered by newly formed tissue that integrated well and appeared similar to the native cartilage tissue [35,36]. In another study by Liu et al. [30], although the study did not evaluate the cartilage repair using the ICRS scoring system, the repair outcome was reported in terms of morphology and integration of the regenerated tissue in the femoral condyle defect. Both HAAM groups (with or without cell seeding with BMSCs) showed new tissue formation; however, the tissue formed in the HAAM + BMSCs group exhibited a more favorable quality compared to the HAAM only group. Specifically, a smooth and surrounding color-matched tissue with good integration was observed in the HAAM-BMSCs group, while a fibrous and less smooth surface was observed in the regenerated tissue from the HAAM-only group. A study by Garcia et al. [31] presented only the final grading of the newly generated tissue. Consistently, all amnion-treated groups showed a grade II “nearly normal” cartilage tissue formation, based on the ICRS macroscopic evaluation, while the control group without any treatment showed a grade III “abnormal” cartilage tissue in the repaired lateral femoral condyle.

**Figure 9:**
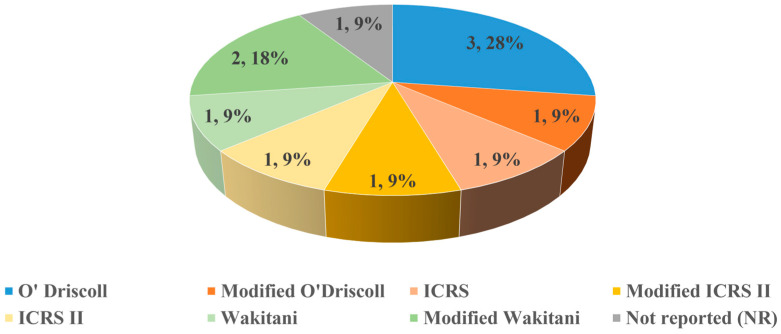
Summary of different scoring systems utilized in the histological analysis in in vivo studies (n = 11). A total of three (3/11, 28%) studies utilized the O’Driscoll scoring system, while the remaining studies employed different histological assessment scores, which include modified O’Driscoll scoring system (1/11, 9%), modified ICRS II (1/11, 9%), ICRS II (1/11, 9%), ICRS (1/11, 9%), Wakitani (1/11, 9%), and modified Wakitani (2/11, 18%) scoring systems. One study (1/11, 9%) reported histological findings without applying any scoring system.

Histological assessment was performed in all 11 in vivo studies. Three (3/11, 28%) utilized the O’Driscoll scoring system, while the remaining studies employed a variety of scales, including modified O’Driscoll scoring system (1/11, 9%), modified ICRS II (1/11, 9%), ICRS II (1/11, 9%), ICRS (1/11, 9%), Wakitani (1/11, 9%), and modified Wakitani (2/11, 18%) scoring systems (Figure 9). Additionally, one study (1/11, 9%) reported histological findings based solely on histopathological staining analysis without applying any scoring system [36]. The predominant histological stains used included Haematoxylin and Eosin (H&E), Safranin-O, Toluidine Blue and Masson Trichrome staining, which helped to characterize the quality and composition of the newly formed cartilage tissue. In addition, for the two ex vivo studies, one reported with ICRS scoring, and one without any scoring system.

In most of the scoring systems, specifically ICRS, ICRS II and O’Driscoll (or their respective modified scoring system), a higher score represents superior tissue quality [41,42,43]. Across these assessment systems, the amnion-treated groups consistently achieved higher scores than the control groups. For instance, a study by Turgut et al. [34], utilizing the modified O’Driscoll scoring system, found that HAM groups consistently achieved higher scores than the untreated control group at both the 4th and 8th week post-treatment. Similarly, Garcia et al. [31] reported that all amnion-treated groups had significantly higher O’Driscoll scores than the untreated control group. Both Tabet et al. [32] and Tabet et al. [33] reported descriptive observations, and they consistently reported that defects treated with amnion were filled with cells resembling hyaline cartilage, in contrast to the untreated control that only showed minimal repair (<10%) or a complete lack of cartilage formation.

Regarding the ICRS II scoring system, Jun et al. [35] observed that the HAAM + JCFs group achieved the highest score among all experimental and control groups. In a study using a modified ICRS II scoring system, Iravani et al. [37] found that the combination of amnion and collagen resulted in significantly better cell morphology and lacuna formation, alongside lower inflammation scores, compared to the untreated control group at both 45- and 90-day post-treatment. While this combination did not differ significantly from the collagen-only scaffold, the HAAM-COL-II composite showed superior cell morphology at day 45 post-treatment. Furthermore, Jin et al. [28] reported that the DHS (denuded HAM with cell seeded on stromal side) group had the highest ICRS score, indicated by complete filling with hyaline cartilage-like tissue, whereas the denuded HAM without cell seeding group showed only partial filling, and the untreated group developed fibrocartilage in the defect created at the patella groove.

Conversely, the Wakitani and modified Wakitani scoring systems utilize a scale where a lower score indicates better tissue regeneration [44]. Cao et al. [29] reported that the ADSCs + AM + PRP group achieved a significantly lower Wakitani score (indicating better cartilage regeneration) compared to all other groups (ADSCs + AM + PRP < ADSCs + AM < ADSCs + PRP < ADSCs). In addition, Liu et al. [30] observed hyaline cartilage formation in the HAAM + BMSCs group, while the HAAM-only group formed scattered chondrocyte-like cells in the repaired femoral condyle, and the untreated control group showed no new cartilage formation. In contrast, Zhang et al. [38] reported that the HAAM-only group had a significantly higher score compared to the HAAM + rBMSCs and the non-defect (normal cartilage) control.

Last but not least, Rastegar Adib et al. [36] reported the repair outcomes without using a scoring system, reporting that the dECM + AME resulted in the most effective cartilage and subchondral bone regeneration. This was evidenced by mature hyaline cartilage stained with intense SO and TB. These findings collectively suggest that the amnion provides a favorable environment for the synthesis of a hyaline-like ECM at the repaired site.

**Figure 10:**
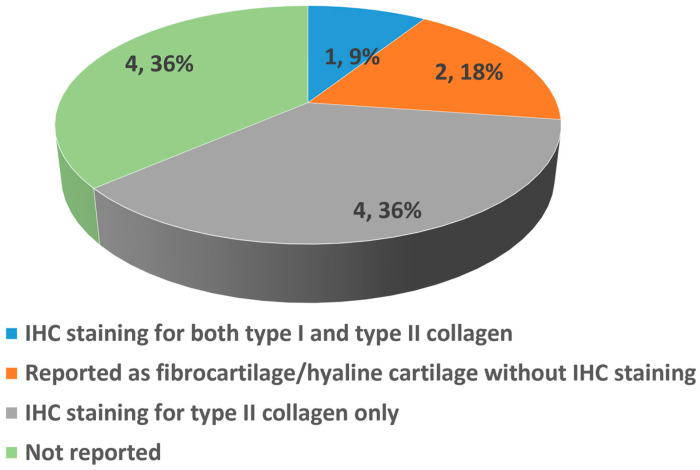
Summary of the expression of type I and type II collagens (COL-I and COL-II) in the repaired cartilage in the in vivo studies (n = 11) based on IHC staining. One study (9%) reported hyaline cartilage repair outcomes with IHC staining for both COL-I and COL-II, while four (36%) studies reported IHC staining for COL-II only. Two (18%) studies reported with descriptive observation without IHC staining and four (36%) studies reported without any IHC results.

IHC analysis was conducted in five out of 11 studies (Figure 10). These studies consistently reported positive results or higher expression of COL-II in the amnion-treated groups compared to other treatment groups. A study by Liu et al. [30] reported that the HAAM + BMSCs group showed positive results for COL-II staining. The studies by Jun et al. [35] and Zhang et al. [38] demonstrated that HAAM utilized with composite or seeded with cells (HAAM + JCFs and HAAM + BMSCs respectively) resulted in a higher content of COL-II compared to the HAAM only and untreated control groups. Additionally, a study by Tabet et al. [33] showed that COL-II was explicitly present in the defects treated with HSAM, whereas it was absent in the untreated control group.

While COL-II is an indicator of high-quality regenerated tissue that represents hyaline cartilage, COL-I commonly serves as a marker for fibrocartilage. Fibrocartilage formation is generally considered an inferior outcome in hyaline cartilage repair. This biological principle is consistent with findings reported across the various anatomical sites included in this review, ranging from the knee articular cartilage examined in most studies (10 out of 11) to the laryngeal cartilage repair investigated using the lamina model [37]. However, one study reported a significantly high expression of both COL-I and COL-II in the amnion-treated cartilage (with the staining intensities highest in the ADSC + AM + PRP group, followed by the ADSC + AM group) [29].

Two studies reported their findings in terms of specific cartilage type (hyaline and fibrocartilage). Jin et al. [28] reported hyaline cartilage formation in the chondrocyte-seeded HAM group, while the untreated defect group showed fibrocartilage formation. Iravani et al. [37] reported progressive IHC evaluation at different time points. At day 45 post-treatment, control defects exhibited predominantly fibrous tissue, while both collagen-only scaffold group and amnion/collagen groups demonstrated superior fibro-hyaline cartilage formation. By day 90 post treatment, this trend had advanced further, with treatment groups achieving predominantly hyaline cartilage characteristics, in contrast to the controls, which exhibited a fibro-hyaline matrix at the repaired thyroid lamina defect. Lastly, four studies assessed neither collagen type nor cartilage type. In studies that did not specify the experimental techniques used to identify cartilage type, the amnion-treated groups generally exhibited hyaline cartilage predominance, whereas control groups exhibited fibrocartilage [28,37].

### 3.8. Application of Amnion Scaffolds for Cartilage Regeneration in Clinical Study

The only clinical study included in this systematic review was conducted by Tabet et al. [39], and the study details are presented in Table 8. This single-arm prospective study involved 10 patients with symptomatic knee cartilage lesions. All patients were treated with a commercially available hypothermically stored amniotic membrane (HSAM) and were monitored for a period of two years following treatment.

At the 24th month of study, all subjects showed significant improvement in KOOS Sports and Recreation and Quality of Life scores, the Marx Activity Scale and the Visual Analog Scale (VAS) for pain. Magnetic Resonance Observation of Cartilage Repair Tissue (MOCART) scoring revealed complete defect repair and filling in seven of the 10 subjects. Three subjects who voluntarily participated in additional biopsy assessments showed integration of the HSAM with native cartilage tissue, and immunohistochemistry staining demonstrated the presence of COL-II throughout the repair site. On the other hand, three subjects experienced mild to moderate treatment-emergent adverse effects (TEAEs), none of which were associated with the HSAM product.

## 4. Discussion

### 4.1. Amnion Procurement, Processing, Composites, and Cell Seeding Strategies

Among the 19 studies included in this review, the majority (n = 17) primarily utilized human amnion as the scaffold source for cartilage regeneration, typically obtained from healthy women undergoing Cesarean sections. In the studies that utilized human amnion, some reported screening donors for infectious diseases, including blood-borne pathogens, but a notable number (n = 5) did not disclose specific procurement criteria. Furthermore, some studies used commercially available human amnion products, which are indeed derived from raw human amnion tissue. Reporting procurement criteria, including donor health status and infectious disease screening, is important to ensure product quality and safety. The lack of uniform procurement criteria raises concerns about potential contamination and disease transmission, which are primary issues in tissue banking [45].

The two studies that utilized animal amnion also failed to report detailed inclusion and exclusion criteria. Procurement criteria are particularly crucial for human amnion because human donors vary significantly in health status, pregnancy conditions and infectious disease exposure. This contrasts with animal amnion procurement, which usually involves controlled laboratory animals with relatively lower biological variability and health risks. Thus, the lack of procurement criteria for animal amnion may be less critical than for human sources. However, to ensure the efficacy and safety of amnion scaffolds in future studies, it is recommended to clearly report all these criteria, regardless of whether the source is lab-prepared human amnion, a commercial amnion product, or animal amnion.

Processing methods varied among the selected studies, including air-drying, lyophilization, cryopreservation, de-epithelialization, and decellularization, as well as the use of fresh and intact amnion. These diverse techniques aim to reduce immunogenicity and improve scaffold integration; however, the absence of standardized protocols hinders direct comparisons across studies. Only one study incorporated collagen into amnion scaffolds [37], showing potential improvements in both mechanical properties and biological activity, thereby fostering a more supportive environment for cartilage repair. There is also a study that utilized an extract from the amnion combined with decellularized sheep osteochondral plugs [36]. Regardless of the processing methods, all amnion-derived scaffolds showed beneficial effects on chondrogenic differentiation and cartilage repair.

To ensure the safety of the amnion scaffold, decontamination and sterilization of the amnion are essential to eliminate potential pathogens prior to application. However, these procedures can impact scaffold bioactivity. Among the 19 studies included, 11 utilized antibiotic and antifungal washes to decontaminate the amnion tissue. While this is a relatively gentle method that preserves the native environment of the amnion, it is considered disinfection or decontamination rather than true sterilization. On the other hand, terminal sterilization processes such as gamma irradiation or ethylene oxide exposure, which are often required for clinical translation, can induce protein denaturation, chemical modification, collagen cross-linking and reduction in essential growth factors [46,47,48]. Hence, optimizing the sterilization dosage is crucial to balance product safety with the preservation of the amnion’s natural properties, which are vital for cartilage repair.

Cell seeding was widely employed to enhance the regenerative potential of amnion scaffolds, with chondrocytes and bone marrow-derived mesenchymal stem cells (BMSCs) being the most commonly used in the selected studies. Other mesenchymal stem cell sources, such as adipose tissue, umbilical cord, and placenta, were used less frequently. This is likely due to chondrocytes being the predominant cell type in mature cartilage [49], while the preference for BMSCs is supported by the evidence suggesting that BMSCs demonstrate superior chondrogenic potential compared to MSCs from other sources in scaffold-free approaches or on various non-amnion scaffolds [50,51]. In addition, amnion has also been reported to be seeded with dental pulp stem cells for evaluating regenerative potential [52]. A limitation of the current literature is the lack of direct comparative evaluations of different types of mesenchymal stem cells seeded on amnion scaffolds. Most of the included studies utilized a single cell source, without parallel comparison to other cell types; only two studies compared different cell types [21,27]. This restricts the ability to determine the cell–scaffold combination for optimized chondrogenic differentiation and cartilage regeneration.

Several studies in this review incorporated composite scaffolds by combining amnion with other biomaterials, including collagen, platelet-rich plasma (PRP), juvenile cartilage fragments (JCFs), and human demineralized bone (DMB), to enhance scaffold functionality. These combinations aimed to improve both the mechanical strength and biological properties of the amnion scaffold. The addition of collagen, a primary structural protein in native cartilage, potentially reinforces the scaffold’s mechanical integrity and provides an enriched microenvironment conducive to cell attachment and differentiation. These composites may better mimic the native cartilage ECM, thereby facilitating improved chondrogenesis and tissue integration. Despite these promising advantages, the limited number of studies utilizing composite scaffolds and the variability in composite formulations highlight the need for more rigorous investigation into optimal composite designs. Future research should focus on systematically comparing composite scaffolds with amnion alone to elucidate their relative benefits in cartilage regeneration applications.

Collectively, these variations in procurement, processing, composite integration, and cell seeding represent critical factors influencing scaffold performance and efficacy in cartilage regeneration. Standardization and thorough reporting of these parameters in future studies are imperative to improve reproducibility, enable meta-analyses, and accelerate clinical translation.

### 4.2. Experiment Models and Defect Characteristics

In the preclinical in vivo studies included in this review, rabbits and sheep are the two main types of animal models used, each offering distinct advantages and limitations. The lapine model is relatively less costly, smaller and easier to handle [53,54]. A drawback of the rabbit model is that it has relatively small joints with thinner cartilage that exhibits an intrinsic spontaneous healing capacity, which may confound regenerative outcomes and limit direct translation to human clinical contexts. On the other hand, sheep possess larger joints and thicker cartilage that more closely resemble human anatomy. This, coupled with limited intrinsic healing ability, makes them a valuable model for clinically relevant cartilage defect studies [55]. However, the larger size and higher activity levels of sheep necessitate more meticulous postoperative management, such as immobilization and scaffold fixation, to prevent scaffold displacement and ensure treatment efficacy. In this context, post-treatment care and immobilization protocols also differ markedly between models. While rabbits are easier to manage with relatively lower immobilization requirements, sheep require stringent postoperative care due to their size and higher mechanical loads, which increase the risk of scaffold displacement.

The defect models employed across studies varied but generally fell into three categories based on the depth of cartilage involvement: partial-thickness defects, which affect only the articular cartilage layer; full-thickness defects, which extend through the calcified cartilage and may or may not expose subchondral bone; and osteochondral defects, which penetrate the subchondral bone. These diverse defect types allow for the investigation of amnion scaffold effects across a spectrum of lesion severities. The commonly used cartilage defect size in rabbit models typically ranges from 3 to 5 mm in diameter, approximately equivalent to 3 to 5 mm in width or length. However, spontaneous healing has been reported in 3 mm diameter defects [56], leading to the recommendation that defects of 4–5 mm diameter are preferred to minimize these confounding effects and improve experimental reliability [57]. Among the reviewed rabbit models, Turgut et al. [34] and Jun et al. [35] used subcritical size defects (3 mm in width and 3.5 mm in diameter, respectively), which may bias outcomes toward actual amnion scaffold efficacy. In contrast, for the sheep models, both studies that specified the dimensions have reached the critical defect size of 7 mm [57]. However, variation in defect size and inconsistent reporting units pose challenges for direct comparison. Standardized reporting of defect dimensions and types would enhance cross-study comparability and strengthen conclusions.

Overall, while small animal models like rabbits are valuable for initial scaffold evaluation due to their cost-effectiveness and ease of handling, their anatomical and functional disparities from human cartilage limit translational relevance [54,58]. Future research should emphasize larger animal models (sheep, goats, and horses) that better approximate human joint biomechanics, cartilage thickness, and defect characteristics, despite inherent challenges such as longer healing times and more complex postoperative management. Harmonizing defect characterization, animal model selection, and reporting standards across studies will enhance the reliability and applicability of preclinical findings on amnion scaffold-mediated cartilage regeneration.

Apart from the choice of animal models and standardized defect reporting, several unique experimental designs were identified. First, the inclusion of biologically distinct cartilage types, such as laryngeal cartilage defects [37], broadens the review’s scope by illustrating the versatility of amnion scaffolds in diverse contexts. However, it also introduces biological heterogeneity, as the composition and regenerative environment of laryngeal cartilage differ from those of articular cartilage. Transparent acknowledgement of these differences and cautious interpretation of such studies’ results are essential to maintain the rigor and contextual relevance of this review.

Second, one study utilized mixed healthy and osteoarthritic samples without inducing lesions on the tissue [22]. In addition, the study failed to separately report outcomes for healthy versus osteoarthritic samples. The absence of a clear distinction between these two groups results in findings that reflect a general interaction between the amnion and human cartilage, rather than definitive conclusions regarding the scaffold’s efficacy across different pathological states.

### 4.3. Limitation of Clinical Evidence

The clinical evidence supporting amnion scaffolds for cartilage repair is currently in its early stages, with only one prospective single-arm study identified, providing preliminary, hypothesis-generating data. This scarcity of human data underscores a critical gap in translating preclinical findings to clinical settings, highlighting the urgent need for more clinical studies evaluating amnion scaffolds for cartilage regeneration. The study by Tabet et al. [39] reported favorable clinical improvements across multiple outcome measures, with no product-related adverse effects observed. While these findings suggest the feasibility and safety of amnion scaffolds for cartilage regeneration, they remain insufficient to establish definitive clinical efficacy over existing treatments.

The utility of the results is constrained by several methodological limitations that compromise their reliability and generalizability. As a single-arm prospective design without randomization or a control group, this study lacks comparators such as conventional treatments (without the use of any scaffolding materials) or using other commercialized biomaterials, or other conservative management. In a nutshell, it remains uncertain whether the observed outcomes resulted specifically from the amnion scaffold or from natural healing processes or placebo effects. Additionally, the small sample size (n = 10), with one patient lost to follow-up after 6 months, further reduces the statistical power and confidence in the reported outcomes. The inclusion of only one clinical study also represents a limitation for this review, preventing comparisons across clinical investigations. Future studies should consider exploring additional search databases, such as Medline, for any potential additional clinical evidence.

Future clinical investigations should prioritize randomized controlled trials (RCTs) comparing amnion scaffolds against established cartilage repair modalities, e.g., the commercialized biomaterials. These trials should be supported by standardized amnion manufacturing processes to ensure product consistency across different studies. In addition, the researchers should set clearly defined clinical indications and utilize harmonized outcome measures such as KOOS and VAS alongside imaging techniques to facilitate cross-study comparisons. Lastly, the implementation of larger sample sizes, longer-term follow-up, and improved strategies to maintain subject retention will also be essential to translating these preliminary findings into evidence-based clinical practice.

## 5. Conclusions

This systematic review provides evidence supporting the efficacy of amnion as a tissue scaffolding material for cartilage regeneration across various in vitro, ex vivo, and in vivo models. However, clinical evidence for the use of amnion in cartilage regeneration remains limited. Future research should therefore prioritize Phase I and Phase II clinical trials. Although amnion has demonstrated promising potential, with several studies reporting superior outcomes compared with other treatment materials, further investigation is required to establish optimized and standardized protocols for amnion scaffold production. This includes focusing on cleanroom-compliant processing and validation in clinical trials before widespread clinical application.

## Figures and Tables

**Table 1:** Study selection criteria used for screening the articles to decide if the articles will be included in this review.

	Inclusion Criteria	Exclusion Criteria
Condition	All in vivo preclinical and clinical studies related to repair of cartilage defects/lesions, i.e., partial, full-thickness, and osteochondral defects, that involves hyaline cartilage from any joints (including knee and larynx); Or, in vitro/ex vivo studies that investigate the use of amnion as a scaffolding material for cartilage tissue engineering.	Use of the osteoarthritis (OA) model.
Interventions	Amnion from any sources (human and animals) as cell delivery scaffold. Scaffold made from any form of amnion (membrane, extract, and powder). Scaffold either with or without cell seeding of any cell type (chondrocytes, mesenchymal stem cells). Scaffold with amnion alone, amnion–chorion or apply in combination with other biomaterials (hydrogel, fibrin glue, collagen, juvenile cartilage fragments, demineralized bone)	Amnion-derived cells (amnion-derived stem cells, exosomes) and amniotic fluid. Only chorion layer.
Outcomes	Result showing chondrogenic differentiation and cartilage regeneration.	Results reported do not relate to cartilage regeneration (wound healing, dental, ophthalmology).
Study Design	Randomized controlled trial, non-randomized controlled trial and prospective study.	
Other		Language other than English.

**Table 2:** List of selected articles (n = 19) included for the systematic review.

Source Article	Article Title	Journal Name/Publication Volume, Page Numbers and Year	Aim of Study
I. ***In vitro* preclinical study**			
Boo et al. [23]	A preliminary study of human amniotic membrane as a potential chondrocyte carrier	Malaysian Orthopaedic Journal. 3(2):16–23. 2009.	To investigate the feasibility of processed human amniotic membrane to support the attachment and proliferation of chondrocytes in vitro.
Krishnamurithy et al. [24]	Human amniotic membrane as a chondrocyte carrier vehicle/substrate: in vitro Study	Journal of Biomaterial Materials Research Part A. (3):500–506. 2011.	To evaluate the feasibility of human amniotic membrane as a chondrocyte carrier by determining if human amnion (HAM) would improve cell proliferation and expression using an in vitro model.
Tan et al. [25]	Human amnion as a novel cell delivery vehicle for chondrogenic mesenchymal stem cells	Cell and Tissue Banking. 12(1):59–70. 2011.	To investigate feasibility of processed human amnion as a substrate for chondrogenic differentiation of mesenchymal stem cells.
Lindenmair et al. [26]	Intact human amniotic membrane differentiated towards the chondrogenic lineage	Cell and Tissue Banking. (2):21–225. 2014.	To evaluate the chondrogenic potential of viable HAM with its sessile cells by in vitro differentiation.
Naseer et al. [27]	Human amniotic membrane as differentiating matrix for in vitro chondrogenesis	Regenerative Medicine. 13(7):821–832. 2018.	To use human amniotic membrane for in vitro chondrogenesis of placenta-derived mesenchymal stem cells and umbilical cord-derived mesenchymal stem cells.
II. ***Ex vivo* study**			
Muinos-Lopez et al. [21]	Human amniotic mesenchymal stromal cells as favorable source for cartilage repair	Tissue Eng Part A. (17–18):901–912. 2017.	To develop an in vitro model for focal articular cartilage using HAM as biomaterial assessing the therapeutic potential of different cell sources.
III. **Both *in vitro* and *ex vivo* study**			
Díaz-Prado et al. [22]	Potential use of the human amniotic membrane as a scaffold in human articular cartilage repair	Cell and Tissue Banking. (2):183–195. 2010.	The aim of this study was to evaluate the potential use of cryopreserved HAMs as human chondrocyte graft support for human articular cartilage repair.
IV. **Both *in vitro* and *in vivo* preclinical study**			
Jin et al. [28]	Human amniotic membrane as a delivery matrix for articular cartilage repair	Tissue Eng. 13(4):693–702. 2007.	To evaluate the feasibility of human amniotic membrane as a chondrocyte carrier by assessing cell proliferation and maintenance of phenotype in vitro and cartilage regeneration in vivo.
Cao et al. [29]	Effect of amniotic membrane/collagen-based scaffolds on the chondrogenic differentiation of adipose-derived stem cells and cartilage repair	Front Cell Dev Biol. 9:647166. 2021.	To investigate the effects of amniotic membrane/collagen scaffolds on the differentiation of adipose-derived mesenchymal stem cells (ADSCs) and articular cartilage repair.
V. ***In vivo* preclinical study**			
Liu et al. [30]	Study of human acellular amniotic membrane loading bone marrow mesenchymal stem cells in repair of articular cartilage defect in rabbits	Genet Mol Res. 13(3):7992–8001. 2014	To investigate the repair effect of human acellular amniotic membrane.
Garcia et al. [31]	Amniotic membrane transplant for articular cartilage repair: An experimental study in sheep	Curr Stem Cell Res Ther. 10(1):77–83. 2015.	To compare the potential for cartilage repair of fresh amnion, cryopreserved amnion and cryopreserved amnion previously culture with bone marrow-derived mesenchymal stem cells (BMSCs).
Tabet et al. [32]	The use of human amniotic membrane for cartilage repair: A sheep study	Stem Cell Discovery. 5(4):40–47. 2015.	To evaluate the human amniotic membrane mixed with demineralized human bone to fill defects in sheep models.
Tabet et al. [33]	The use of hypothermically stored amniotic membrane for cartilage repair: A sheep study	Stem Cell Discovery. 5(4):62–71. 2015.	To evaluate the use of hypothermically stored human amniotic membrane for cartilage repair in adult sheep.
Turgut et al. [34]	The effects of human amniotic fluid and membrane on chondral healing in a rabbit knee cartilage defect model	Medical Journal of Süleyman Demirel University. 28(4):663–671. 2021.	To determine the effects of human amniotic fluid and membrane on chondral defects.
Jun et al. [35]	Human acellular amniotic membrane scaffolds encapsulating juvenile cartilage fragments accelerate the repair of rabbit osteochondral defects	Bone Joint Res. 11(6):349–361. 2022.	To explore the effect of human acellular amniotic membrane scaffolds with juvenile cartilage fragments on osteochondral defects.
Rastegar Adib et al. [36]	Osteochondral regeneration in rabbit using xenograft decellularized ECM in combination with different biological products; platelet-rich fibrin, amniotic membrane extract, and mesenchymal stromal cells	Journal of Biomedical Materials Research Part B Applied Biomaterials. 110 (9):2089–2099. 2022.	To investigate the regenerative effect of decellularized osteochondral ECM xenograft in combination with various biological products in an osteochondral defect.
Iravani et al. [37]	Effect of amniotic membrane/collagen scaffolds on laryngeal cartilage repair	Laryngoscope Investig Otolaryngol. 9(1):e1222. 2024.	To evaluate the efficacy of a collagen scaffold enveloped by amniotic membrane on laryngeal cartilage repair.
Zhang et al. [38]	Amniotic membrane derived-stem cells help repair osteochondral defect in a weight-bearing area in rabbits	Exp Ther Med. (1):187–192. 2017.	To evaluate the effects of human acellular amniotic membrane seeded with bone marrow-derived mesenchymal stem cells for repairing osteochondral defects in a weight-bearing area in rabbits.
VI. **Clinical study**			
Tabet et al. [39]	Hypothermically stored amniotic membrane for the treatment of cartilage lesions: A single-arm prospective study with 2-year follow-up	Cartilage. 13(1):19476035211072213. 2022.	To determine the safety and efficacy of hypothermically stored amniotic membrane for the treatment of knee cartilage lesions.

**Table 3:** Amnion procurement criteria and preparation in the selected articles (with and without cell seeding).

Reference	Source of Amnion	Screening Criteria for Amnion Procurement		Preservation/Processing Methods	Type of Composite (if Applicable)	Type of Cells Seeded
		Inclusion	Exclusion			
**Without Cell Seeding on Amnion**						
Lindenmair et al. [26]	Human	An individual who underwent a cesarean section	NR	Fresh/Intact	Only amnion	Without cell seeding
Tabet et al. [32]	Human	NR	NR	Fresh/NR	With demineralized human bone	Without cell seeding
Tabet et al. [33]	Human (commercially available amnion)	NA	NA	Hypothermic storage in AlloFresh™ solution/NR	Only amnion	Without cell seeding
Turgut et al. [34]	Human	Seronegative parturient	NR	Fresh amnion/NR	Only amnion	Without cell seeding
Jun et al. [35]	Human	NR	NR	Fresh/De-epithelialization	With and without juvenile cartilage fragments (JCFs)	Without cell seeding
Rastegar Adib et al. [36]	Human	From placenta of the healthy women	NR	NR/Cryo-pulverization and extraction	With decellularized extracellular matrix	Without cell seeding
Iravani et al. [37]	Human	Women who did not have a history of pregnancy problem and had undergone elective caesarean section	NR	Cryopreservation/NR	With collagen	Without cell seeding
Tabet et al. [39]	Human (commercially available amnion)	NA	NA	Hypothermic storage in AlloFresh™ solution/NR	Only amnion	Without cell seeding
**With cell seeding on amnion**						
Boo et al. [23]	Human and commercially available glycerol-preserved human amnion	Caesarean-sectioned mothers who were seronegative for HBV, HCV, syphilis and HIV	NR	Air-dried, lyophilization, and glycerol preservation/NR	Only amnion	Seeded with rabbit autologous chondrocytes
Krishnamurithy et al. [24]	Human	The individual underwent caesarean-section, age within 25–35 years old, and negative for HBC, HCV, syphilis, and HIV	NR	Air-dried and freeze-dried/NR	Only amnion	Seeded with rabbit chondrocytes
Tan et al. [25]	Human	Individuals who underwent elective caesarean sections and were seronegative for HIV, HBV, HCV, and syphilis	NR	Air-dried and lyophilization/NR	Only amnion	Seeded with rabbit BMSCs
Naseer et al. [27]	Human	Full-term caesarean section mothers who are HIV, HBV, and HCV negative and without complications during pregnancy	NR	Cryopreservation/De-epithelialization	Only amnion	With human PMSCs and human UMSCs
Muinos-Lopez et al. [21]	Human	Healthy donor underwent elective caesarean Sections with informed consent	NR	Cryopreservation/NR	Only amnion	Seeded with human articular chondrocytes, human BMSCs, human amniotic epithelial cells, human amniotic MSCs vs. control (amnion without cell seeding)
Díaz-Prado et al. [22]	Human	Selected caesarean-sectioned mothers with informed consent	NR	Cryopreservation/De-epithelialization	Only amnion	Seeded with human chondrocytes vs. control (amnion without cell seeding)
Jin et al. [28]	Human	30–35 years old caesarean-sectioned mother and negative for HBV, HCV, syphilis and HIV	NR	Cryopreservation/Intact and de-epithelialization	Only amnion	With rabbit chondrocytes
Cao et al. [29]	Rabbit	NR	NR	Lyophilization/Decellularization	With PRP	Seeded with rabbit ADSCs, and amnion without cell seeding
Liu et al. [30]	Human	From healthy parturient	Positive for HIV, HBV, HCV or syphilis	NR/De-epithelialization	Only amnion	Seeded with BMSCs vs. amnion without cell seeding
Garcia et al. [31]	Sheep	NR	NR	Fresh and cryopreservation/NR	Only amnion	Cryopreserved amnion seeded with sheep BMSCs, fresh amnion without cell seeding and cryopreserved amnion without cell seeding
Zhang et al. [38]	Human	NR	NR	NR/Decellularization	Only amnion	Seeded with rabbit BMSCs vs. amnion without cell seeding

Acronym: ADSCs: Adipose-derived mesenchymal stem cells; BMSCs: Bone marrow-derived mesenchymal stem cells; HBV: Hepatitis B virus; HCV: Hepatitis C virus; HIV: Human immunodeficiency virus; NA: Not applicable; NR: Not reported; PMSCs: Placenta-derived mesenchymal stem cells; PRP: Platelet-rich plasma; and UMSCs: Umbili-cal-cord-derived mesenchymal stem cells.

**Table 4:** In vitro preclinical studies from the selected articles.

Reference	Source of Amnion	Type of Cells Tested/Source	Grouping/Study Duration (Day/Week)	Findings	
				Cell/Tissue Morphology, Viability, Proliferation and Attachment	Cartilage Related Marker Expression
***Without cell seeding on amnion***					
Lindenmair et al. [26]	Human	Without cell seeding	G1: Control medium G2: Chondrogenic medium (C) G3: Chondrogenic medium with fibroblast growth factor 2 (FGF2) (C-FGF) G4: Chondrogenic redifferentiation medium (T) Study duration: 56 days	Chondrocyte redifferentiation medium (T) sustained the highest viability (56.2 ± 10.5%) with high number of cells in epithelial layer. Chondrogenic media (C and C-FGF) caused a rapid early decline (24.6 ± 2.4% and 21.7 ± 4.4%, respectively). These media supported growth primarily for cells in mesenchymal layer. Control media (CM) resulted in the lowest final viability at day 56 (15.1 ± 2.2%), in which cells in both.	Both chondrogenic media (C and C-FGF) groups showed a significant increase in the glycosaminoglycan (GAG)/viability ratio compared to day 0, reaching peak value at day 56 (20.60 ± 8.93, *p* < 0.01; 29.88 ± 0.89, *p* < 0.001, respectively). The control and redifferentiation groups showed no significant difference from the baseline (11.27 ± 1.27, 7.47 ± 2.47). Type II collagen was locally detected only in chondrogenic groups (C and C-FGF, with C-FGF showing more areas of staining), while type I collagen remained uniform throughout the matrix, and collagen type X was not detected in any condition. Chondrogenic media (C and C-FGF) and chondrocyte redifferentiation medium (T) upregulated the cartilage-related genes COMP, CSPG2, COL1A1, COL9A2, MIA, and CRTL1, whereas SOX9 was downregulated across all conditions.
***With cell seeding on amnion***					
Boo et al. [23]	Human	Rabbit chondrocytes seeded on basement layer	G1: Air-dried human amnion (AD HAM) G2: Freeze-dried human amnion (FD HAM) G3: (Glycerol preserved human amnion) GPHAM Study duration: 21 days	Chondrocytes in all amnions showed proliferation. AD HAM and FD HAM showed some cell detachment during medium changes, while almost all cells attached to GPHAM.	NA
Krishnamurithy et al. [24]	Human	Rabbit chondrocytes seeded on basement layer	G1: AD HAM + rabbit chondrocytes G2: FD HAM + rabbit chondrocytes G3: Rabbit chondrocytes cultured on monolayer Study duration: 28 days	Cell proliferation in both AD HAM (13–51%, *p* = 0.001) and FD HAM (18–48%, *p* = 0.001) are significantly higher than in the monolayer, but no significant difference between AD HAM and FD HAM (*p* = 0.576). Chondrocytes attached to both AD HAM and FD HAM, exhibiting large, dense, ovoid, and centrally located nuclei.	AD HAM and FD HAM showed significant increase in total GAG as compared to monolayer cultures from day 3 to 28. There was no significant difference in GAG content per cell between than AD HAM and FD HAM. SEM analysis showed that chondrocytes formed continuous fusiform layers on the smooth AD HAM surface, while FD HAM supported large cell colonies within its porous structure.
Tan et al. [25]	Human	Rabbit BMSCs	G1: Control (rBMSCs in monolayer) G2: Negative control (HAM without cells) G3: AD HAM + rBMSCs G4: FD HAM + rBMSCs Study duration: 15 days	Cells were found to attached on both HAM but not in the negative control (HAM without cell seeding).	HAM showed a statistically significant increase in GAG expression compared to the monolayer control, while there was no increase in GAG observed in the negative control group. (AD HAM: 0.93 to 1.31, FD HAM: 0.99 to 1.69, monolayer: 0.21 to 0.68)
Naseer et al. [27]	Human	Human placenta-derived MSCs and human umbilical cord-derived MSCs	G1: HAM + PMSCs (Differentiated cells) G2: HAM + UMSCs (Differentiated cells) G3: HAM + PMSCs (Control cells) G4: HAM + UMSCs (Control cells) G5: Plastic surface + PMSCs (Differentiated cells) G6: Plastic surface + UMSCs (Differentiated cells) G7: Plastic surface + PMSCs (Control cells) G8: Plastic surface+ UMSCs (Control cells) Study duration: 14 days	PMSCs and UMSCs underwent morphological changes from fibroblast-like to polygonal or rounded shapes on both plastic and HAM by day 14, with PMSCs specifically exhibiting cell aggregation and binucleation.	Safranin-O staining and Image J quantification confirmed significantly increased proteoglycan content in both PMSCs- and UMSCs-derived chondrocyte-like cells on plastic and HAM compared to control groups. Treated PMSCs and UMSCs on both plastic and HAM demonstrated increased expression of type II collagen and aggrecan compared to the untreated control group.
Díaz-Prado et al. [22]	Human	Human chondrocytes seeded on basement layer	G1: Chondrocytes seeded on epithelial layer G2: Chondrocytes seeded on basement layer Study duration: 16 weeks	Chondrocytes grew in a characteristic monolayer pattern on both the epithelial and basement sides, but eosinophilia, massive necrosis, fragmentation and detachment of the chondrocytes were observed in the epithelial side of the HAM.	Type II collagen was detected, while type I collagen was absent in chondrocytes cultured on the HAM basement membrane.
Jin et al. [28]	Human	Rabbit chondrocytes	G1: Positive control (chondrocyte before seeding) G2: Negative control (intact HAM without cells) G3: Chondrocytes seeded on intact HAM epithelial side (IHE) G4: Chondrocytes seeded on denuded HAM basement side (DHB) G5: Chondrocytes seeded on denuded HAM stromal side (DHS) Study duration: 4 weeks	High cell attachment rates were observed on all HAM substrates without a significant difference. Cell viability was maintained in all HAM substrate without apparent stain of dead cells.	IHC: Type II collagen was detected in the DHS group with accumulation supported by the Western blot analysis result. Type II collagen in DHB was only detected via Western blot analysis with declining trend. Type II collagen was not detected in IHE.
Cao et al. [29]	Rabbit	Rabbit ADSCs	G1: Control (ADSCs) G2: ADSCs + PRP G3: ADSCs + AM G4: ADSCs + AM + PRP Study duration: 21 days	ADSC proliferation and viability, along with the expression of key chondrogenic genes and proteins, showed a consistent pattern with the highest levels in the amniotic membrane + PRP + ADSCs group, followed by amniotic membrane + ADSCs, then PRP + ADSCs, and lowest in the ADSCs-only control group. Distinct cartilage formation was observed only in the amniotic membrane + PRP group by light microscopy	Type II collagen expression predominated over types I and X.

Acronym: AD HAM: Air-dried human amnion; ADSCs: Adipose-derived mesenchymal stem cells; COL1A1: Collagen type I alpha 1 chain, COL9A2: Collagen type IX alpha 2 chain; COMP: Cartilage oligomeric matrix protein; CRTL1: Cartilage link protein 1; CSPG2: Chondroitin sulfate proteoglycan 2; DHB: Denuded HAM basement side; DHS: Denuded HAM stromal side; FD HAM: Freeze-dried human amniotic membranes; GAG: glycosaminoglycan; GPHAM: Glycerol-preserved intact human amniotic membranes; IHC: Immunohistochemistry; IHE: Intact HAM epithelial side; MIA: Melanoma Inhibitor Activity; PMSCs: Placenta-derived mesenchymal stem cells; PRP: Platelet-rich plasma; rBMSCs: Rabbit bone marrow-derived mesenchymal stem cells; SOX9: SRY (Sex Determining Region Y)-Box 9; and UMSCs: Umbilical-cord-derived mesenchymal stem cells.

**Table 5:** Ex vivo and in vivo preclinical studies from the selected articles (small and large animal models).

Reference	Animal Species (Sample Size)	Defect Model/Dimension	Defect Creation Method/Location
***Ex vivo* study**			
Díaz-Prado et al. [22]	Human cartilage biopsies (N = 48)	Human articular cartilage biopsies were cut into 6 mm diameter disc	Without defect creation
Muinos-Lopez et al. [21]	Human cartilage biopsies (N > 22)	Human cartilage biopsies were cut using biopsy punch into 6 mm diameter with 2 mm diameter focal lesion	Used dental drill to create defect in the superficial zone of the cartilage biopsies
***In vivo* study**			
**(*i*)** **Small animal model—Lapine model**			
Jin et al. [28]	New Zealand white rabbits (N = 12)	Osteochondritis defect (Diameter 5 mm)	Used 5 mm drill to create defect at patella groove
Iravani et al. [37]	Dutch rabbits (N = 14)	Symmetric cartilage defects (Diameter 5 mm)	The defects were created at both sides of the thyroid lamina
Cao et al. [29]	New Zealand white rabbits (NR)	Articular cartilage defect (NR)	Used sharp instrument to create the defect.
Liu et al. [30]	New Zealand rabbits (N = 24)	Bilateral full-thickness cartilage defects (Diameter 4 mm × depth 3 mm)	Used 4 mm drill bit to create defect on bilateral femoral condyle
Turgut et al. [34]	Albino New Zealand rabbits (N = 32)	Bilateral full-thickness cartilage defect without damaging the subchondral bone (Width 3 mm × length 7 mm)	Used scalpel to create defect on medial femoral condyle
Jun et al. [35]	New Zealand rabbits (N = 20)	Osteochondral defect (Diameter 3.5 mm × depth 3 mm)	Used dental drill to create the defect in the centre of the femoral groove
Rastegar Adib et al. [36]	New Zealand white rabbits (NR)	Osteochondral defect (3.5 diameter and 5 mm depth)	Used stainless steel trephine drilling to create defects in the femoral trochlear groove of both the left and right knee
Zhang et al. [38]	New Zealand white rabbits (N = 24)	Bilateral osteochondral defects (depth: 3 mm)	Used 4 mm drill to create defect bilaterally at medial femoral condyle
**(*ii*)** **Large animal model—Ovine model**			
Tabet et al. [33]	Suffolk-cross ewes (N = 5)	Partial-thickness cartilage defect (1 cm^2^)	Used curette to create defect at trochlear
Garcia et al. [31]	Ovis aries sheep (N = 12)	Full-thickness cartilage defect without involving the subchondral bone (7 × 5 mm cm^2^)	Used scalpel and sharp spoon to create defect on the lateral femoral condyle
Tabet et al. [32]	Sheep (N = 6)	NR	Used curette to create two defects on the same knee: one on the femoral condyle and another in the trochlear groove

Acronym: NR—Not reported.

**Table 6:** Various types of in vivo cartilage defect models reported in the preclinical studies (n = 11). (**a**) Defect in the shape of a circle; (**b**) defect in the shape of a square.

**(a) Defect in the Shape of a Circle**				
**Animal Breed**	**Defect Diameter (mm)**	**Defect Depth (mm)**	**Area (cm^2^)**	**Reference**
New Zealand white rabbits	NR	3	NR	Zhang et al. [38]
New Zealand rabbits	3.5	3	NR	Jun et al. [35]
New Zealand white rabbits	3.5	5	NR	Rastegar Adib et al. [36]
New Zealand rabbits	4	3	NR	Liu et al. [30]
New Zealand white rabbits	5	NR	NR	Jin et al. [28]
Dutch rabbits	5	NR	NR	Iravani et al. [37]
New Zealand white rabbits	NR	NR	NR	Cao et al. [29]
Sheep	NR	NR	NR	Tabet et al. [32]
**(b) Defect in the shape of a square**				
**Animal breed**	**Defect width (mm)**	**Defect length (mm)**	**Area (cm^2^)**	**Reference**
Albino New Zealand white rabbits	3	7	NR	Turgut et al. [34]
*Ovis aries* sheep	7	5	NR	Garcia et al. [31]
Suffolk-cross ewes	NR	NR	1	Tabet et al. [33]

Acronym: NR—Not reported.

**Table 7:** Morphological, histological and immunohistochemical evaluation of cartilage repair using amnion in ex vivo (n = 2) and in vivo (n = 11) studies.

Reference	Source of Amnion	Type of Cell Tested	Grouping/Study Duration	Findings		
				Gross Findings	Histological Findings	IHC/Type of Cartilage Formed
***Ex vivo* study**						
**Díaz-Prado et al.** [22]	Human	Human chondrocytes seeded on basement layer	G1: control (only HAM) G2: HAM with chondrocytes Study duration: 16 weeks	NR	In the control group, the amniotic membrane adhered to the cartilage but failed to generate any new tissue. The newly formed tissue in HAM seeded with chondrocyte showed good integration with the native cartilage, but Safranin-O staining was negative in nearly all cases, indicating a lack of proteoglycans in the regenerated tissue.	The newly formed tissue in the HAM-chondrocyte group showed a positive reaction for type II collagen, whereas type I collagen expression was weak or absent.
**Muinos-Lopez et al.** [21]	Human	With human articular chondrocytes, human BMSCs, human amniotic epithelial cells, human amniotic MSCs seeded on the stromal layer	G1: control (only HAM) G2: HAM with human chondrocytes G3: HAM with hBMSCs G4: HAM with hAECs G5: HAM with hAMSCs Study duration: 8 weeks	NR	Chondrocytes, hBMSCs, hAMSCs, and hAECs did not show significant differences in the ICRS scoring. HAM with chondrocytes showed the best quality of integration with the native cartilage. Chondrocytes and hAMSCs showed metachromasia for both stains when compared with hBMSCs, while hAECs showed negative for safranin-O (SO) and only slightly positive for toluidine blue (TB) stain.	The type II collagen content was higher in hAMSCs compared to other cells, and was significantly higher compared with chondrocytes. All cell types showing the presence of type I collagen content, with the content found in the hBMSCs group significantly higher than in other groups.
***In vivo* study**						
Jin et al. [28]	Human	Rabbit chondrocytes	G1: null (no amnion applied) G2: denuded HAM with stromal layer facing the defect G3: denuded HAM stromal side (DHS) with seeded cells Study duration: 8 weeks	NR	The DHS group had the highest ICRS score (15.75 ± 1.71, *p* < 0.001). The DHS group showed complete defect filling with fully mature cartilage that resembles native hyaline cartilage. The null group form mainly fibrocartilage, and the denuded HAM group had incomplete regeneration with partial defect filling at 8th week.	G1: fibrocartilage G2: NR G3: hyaline cartilage
Cao et al. [29]	Rabbit	ADSCs	G1: control (culture medium) G2: ADSCs G3: AM G4: ADSCs + AM G5: ADSCs + platelet-rich plasma (PRP) G6: ADSCs + AM + PRP Study duration: 12 weeks	NR	Wakitani scores of the ADSC + AM + PRP group (1.33 ± 0.32) were significantly lower compared to other groups. The ADSC + AM (2.63 ± 0.38) group had the second-lowest Wakitani score, followed by the ADSC + PRP (4.4 ± 0.44) group and ADSCs group (6.733 ± 0.21).	IHC analysis revealed that the level of type I collagen and type II collagen in the ADSC + AM + PRP group were the highest, followed by the ADSC + AM group. Both had significantly higher levels of type I collagen and type II compared to all other groups.
Liu et al. [30]	Human	BMSCs	G1: Human acellular amniotic membrane (HAAM) + BMSCs G2: only HAAM Right-side defects in each group were used as controls. Study duration: 12 weeks	At 12 weeks, group 1’s defect area had smooth new tissue that matched the surrounding normal cartilage in color and integrated well. In group 2, the newly formed tissue was milky white, fibrous, with a less smooth surface, and hard texture.	Modified Wakitani Scoring Group 1: formed mainly hyaline cartilage-like cells, with much cartilage-like matrix with normal colouring. Group 2: Scattered cartilage-like cells. Control: no cartilage-like cells or tissue was visible.	Group 1 showed a positive result for type II collagen IHC staining.
Garcia et al. [31]	Sheep	Sheep BMSCs seeded on stromal layer	G1: Control G2: Fresh amnion G3: Cryopreserved amnion previously cultured with BMSCs G4: Only cryopreserved amnion Study duration: 2 months	Control group showed abnormal ICRS grade (grade III) while other treatment groups showed normal ICRS grade (grade II).	Control group shows significantly lower score compared to all the treatment groups based on O’Driscoll scale. (G1: 3.33, G2: 10.66, G3: 8, G4: 11.33)	NR
Tabet et al. [32]	Human	Without cell seeding, with stromal layer facing the defects	G1: Control G2: HAM/Demineralized bone (DMB) Study duration: 6 months	NR	O’Driscoll Grading Scale was used. Control group: the defects did not fill with either type of cartilage. The defects with retained membranes showed diffuse proliferation of chondrocyte-like cells within a stromal matrix resembling hyaline cartilage.	NR
Tabet et al. [33]	Human	Without cell seeding, with stromal layer facing the defects	G1: HSAM G2: Defect control (defect without treatment) G3: Normal control (without defect) Study duration: 5 months	NR	O’Driscoll Grading Scale was used. HSAM group: showed nearly complete defect fill with abundant cartilage-like cells within a stromal matrix resembling hyaline cartilage, exhibiting strong integration with the surrounding host cartilage. Defect control group: demonstrated minimal repair, with less than 10% fill.	Based on IHC staining, type II collagen is shown in the HSAM group but not in the defect control group.
Turgut et al. [34]	Human	Without cell seeding	G1: Sham control G2: Human amniotic fluid (HAF) G3: HAM G4: HAM + HAF Study duration: 12 weeks	NR	For Modified O’Driscoll Grading Scale at 4th week: Compared to sham control average score, HAF was slightly lower, while HAM and HAM + HAF had slightly higher scores. (G1: 6.750± 1.035, G2: 6.625± 2.066, G3: 7.100± 2.558, G4: 7.200± 2.201) At 8th week: Compared to sham control average score, HAM was slightly higher, while HAM + HAF was slightly lower. HAF had a similar score to sham control. (G1: 4.625 ± 1.408, G2: 4.625 ± 2.446, G3: 4.833 ± 1.329, G4: 4.000± 1.414)	NR
Jun et al. [35]	Human	Without cell seeding	G1: Control G2: HAAM scaffold G3: Juvenile cartilage fragments (JCFs) G4: HAAM + JCFs Study duration: 12 weeks	The HAAM + JCFs group had the highest ICRS score, indicated by the newly formed cartilage that was similar to native cartilage, completely covered the defects, and showed good integration with native cartilage.	For ICRS II, the HAAM + JCFs group had a significantly higher score compared to other groups, the result of all staining was superior to other three groups.	Immunohistochemistry (IHC) for type II collagen revealed the greatest amount of type II collagen in the HAAM + JCFs group, followed by the HAAM and JCFs groups. The control group showed negative staining in the IHC.
Rastegar Adib et al. [36]	Human	Without cell seeding	G1: Control without treatment G2: dECM only G3: dECM + Platelet-rich fibrin (PRF) G4: dECM + AME G5: dECM + rBMSCs Study duration: 12 weeks	The ICRS macroscopic scoring showed that adding biological products to dECM greatly improved the repair, with the combination involving AME providing the best result (100%), where the lesion fully healed with smooth surface resembling normal cartilage with good integration.	dECM + AME (86.5 ± 5.9%) resulted in the best cartilage and subchondral bone regeneration with mature hyaline cartilage evidenced by strong SO and TB staining, whereas the control and dECM group (40.5 ± 8.3%) showed fibrocartilage and immature cartilage formation.	NR
Iravani et al. [37]	Human	Without cell seeding	G1: control without treatment G2: collagen scaffolds G3: amnion and collagen scaffolds (AM/C) Study duration: 90 days	NR	Modified ICRS II scoring was used. AM/C group have significantly higher score of cell morphology, lacuna formation and lower inflammation compared to the control at both the 45th and 90th day. (cell morphology: 1.75 ± 0.50 vs. 1.14 ± 0.38; 2.50 ± 0.71 vs. 1.33 ± 0.52, p < 0.05). (inflammation: 1.50 ± 0.58 vs. 2.86 ± 0.69; 0.50 ± 0.71 vs. 2.17 ± 0.75, p < 0.05) Except for cell morphology criteria at the 45th day, AM/C has no significant difference compared to pure collagen scaffold in all histological parameters.	At day-45: the control group shows more fibrous tissue, while both treatment groups show more fibro-hyaline cartilage formation At day-90: the control group shows more fibro-hyaline cartilage, while both treatment groups show more hyaline cartilage formation.
Zhang et al. [38]	Human	With or without rabbit BMSCs	G1: control (without defect and treatment) G2: HAAM G3: HAAM with rBMSCs Study duration: 24 weeks	NR	Modified Wakitani score: the control group is significantly lower than other two groups, the HAAM-rBMSCs group is still significantly low compared to only HAAM group. (week 12: 0.00 ± 0.01 vs. 6.33 ± 0.38 vs. 10.38 ± 0.21, *p* < 0.05; week 24: 0.00 ± 0.01 vs. 3.05 ± 1.28 vs. 9.47 ± 1.11, *p* < 0.05). HAAM-rBMSCs group: score for 24th week is significantly lower than 12th week (3.05 ± 1.28 vs. 6.33 ± 0.38, *p* < 0.05). HAAM group: no significant difference between 12th and 24th week (10.38 ± 0.21 vs. 9.47 ± 1.11, *p* > 0.05). H&E: the tissue coverage in the HAAM-BMSCs group is significantly higher than the HAAM group but showed no significant difference with the control. Toluidine blue: the number of chondrocytes in the HAAM-rBMSCs group is significantly higher than in the HAAM group but showed no significant difference with the control.	Expression of type II collagen in the HAAM-rBMSCs group was significantly higher than in the only HAAM. No significant difference between the control and HAAM-rBMSCs.

Acronym: ADSCs: Adipose-derived mesenchymal stem cells; AM: amnion; AME: Amnion extract; dECM: decellularized ECM; DHS: Denuded HAM stromal side; DMB: Demineralized bone; HAAM: Human acellular amniotic membrane; hAECs: Human amniotic epithelial cells; HAF: Human amniotic fluid; HAM: Human amnion; hAMSCs: Human amniotic MSCs; hBMSCs: Human bone marrow-derived mesenchymal stem cells; HSAM: Hypothermically stored amniotic membrane; ICRS: International Cartilage Repair Society; IHC: Immunohistochemistry; JCFs: Juvenile cartilage fragments; NR—Not reported; PRF: Platelet-rich fibrin; PRP: Platelet-rich plasma; and rBMSCs: Rabbit bone marrow-derived mesenchymal stem cells.

**Table 8:** Clinical study from the selected articles of Tabet et al. [39].

Clinical Condition	Symptomatic Cartilage Lesions in the Knee
Patient inclusion/exclusion criteria	Inclusion criteria: Age: 18–55 years old Symptomatic, focal, contained chondral lesions (ICRS grade 3/grade 4A of the femur), with defect areas (1–5 cm2) after debridement At least 3 months post-surgery of any previous surgeries on the study knee with an asymptomatic, stable and fully functional contralateral knee Exclusion criteria: BMI > 35 kg/m^2^ Presence of bipolar lesions/kissing lesions of ipsilateral compartment prior total meniscectomy of either knee failed microfracture within 12 months of surgery Radiographic malalignment greater than 5° measured from the hip, knee and ankle mechanical axis Patients who were diagnosed with osteoarthritis, rheumatoid arthritis, gout or avascular necrosis
Sample size	10 enrolled (but 1 lost to follow-up after 6 months)
Study duration	24 months
Clinical condition	Symptomatic cartilage lesions in the knee
Grouping/Test conditions	Only 1 group (n = 10) All patients received treatment of the commercially available hypothermically stored amniotic membrane (HSAM) with stromal layer facing the defects
Source of amnion	Human
Type of cells Tested/Source	NR
Culture condition	NR
Findings	KOOS Sports & Recreation and Quality of Life improved from baseline to 24 months Marx Activity Scale improved from 12 to 24 months VAS improved from baseline to 24 months MOCART scoring showed 7/10 subjects had complete defect repair and filling by 24 months Showed integration of HSAM with the native cartilage and type II collagen is detected along the repair site
Complications	3 subjects had reported at least one mild to moderate adverse event but none of them is related to the HSAM

Acronym: BMI: Body mass index; HSAM: Hypothermically stored amniotic membrane; ICRS: International Cartilage Repair Society; KOOS: Knee Injury and Osteoarthritis Outcome Score; MOCART: Modified Magnetic Resonance Observation of Cartilage Repair Tissue; NR: Not reported; and VAS: Visual Analogue Scale.

## Data Availability

The original contributions presented in the study are included in the article, further inquiries can be directed to the corresponding authors.

## Abbreviations

The following abbreviations are used in this manuscript:

AD HAM	Air-dried human amnion
ADSCs	Adipose-derived mesenchymal stem cells
AECs	Amniotic epithelial cells
AM	Amnion
AME	Amnion extract
AMSCs	Amnion-derived mesenchymal stem cells
BMI	Body mass index
BMSCs	Bone marrow-derived mesenchymal stem cells
COL-I	Type I collagen
COL-II	Type II collagen
COL1A1	Collagen type I alpha 1 chain
COL9A2	Collagen type IX alpha 2 chain
COMP	Cartilage oligomeric matrix protein
CRTL1	Cartilage link protein 1
CSPG2	Chondroitin sulfate proteoglycan 2
dECM	decellularized ECM
DHB	Denuded HAM basement side
DHS	Denuded HAM stromal side
DMB	Demineralized bone
ECM	Extracellular matrix
FD HAM	Freeze-dried human amniotic membranes
FGF	Fibroblast growth factor
GAG	Glycosaminoglycan
GPHAM	Glycerol-preserved intact human amniotic membranes
hAECs	Human amniotic epithelial cells
HAF	Human amniotic fluid
HAAM	Human acellular amniotic membrane
HAM	Human amnion (human amniotic membranes)
hAMSCs	Human amniotic MSCs
hBMSCs	Human bone marrow-derived mesenchymal stem cells
HBV	Hepatitis B virus
HCV	Hepatitis C virus
HIV	Human immunodeficiency virus
HSAM	Hypothermically stored amniotic membrane
ICRS	International Cartilage Repair Society
IHC	Immunohistochemistry
IHE	Intact HAM epithelial side
JCFs	Juvenile cartilage fragments
KOOS	Knee Injury and Osteoarthritis Outcome Score
MIA	Melanoma Inhibitor Activity
MINORS	Methodological Index for Non-Randomized Study
MOCART	Modified Magnetic Resonance Observation of Cartilage Repair Tissue
MRI	Magnetic resonance imaging
MSCs	Mesenchymal stem cells
NR	Not reported
NA	Not applicable
OA	Osteoarthritis
OHAT	Office of Health and Translation
PMSCs	Placenta-derived mesenchymal stem cells
PRISMA	Preferred Reporting Items for Systematic Reviews and Meta-Analyses
PRF	Platelet rich fibrin
PROSPERO	Prospective register of Systematic Review
PRP	Platelet-rich plasma
QoL	Quality of Life
rBMSCs	Rabbit bone marrow-derived mesenchymal stem cells
RCTs	Randomized controlled trials
SO	Safranin-O
SOX9	SRY (Sex Determining Region Y)-Box 9
TB	Toluidine blue
TEAEs	Treatment-emergent adverse events
UMSCs	Umbilical-cord-derived mesenchymal stem cells
VAS	Visual Analogue Scale

## Appendix A

Table A1 shows the detailed search strategies for the main databases utilized for this systematic review.

**Table A1:** Search strategies of three different databases, PubMed, Web of Science (WoS) and Scopus.

Database	Search String *
PubMed	((“full-thickness cartilage defect”[Title/Abstract] OR “cartilage damage”[Title/Abstract] OR “cartilage lesions”[Title/Abstract] OR “cartilage defects”[Title/Abstract] OR “cartilage injury”[Title/Abstract] OR “cartilage injuries”[Title/Abstract] OR “cartilage destruction”[Title/Abstract] OR “cartilage deterioration”[Title/Abstract] OR “chondral lesion”[Title/Abstract] OR “articular cartilage injury”[Title/Abstract] OR “osteochondral defects”[Title/Abstract] OR “cartilage, articular”[MeSH Terms] OR “cartilage diseases”[MeSH Terms]) AND (“human amnion”[Title/Abstract] OR “amniotic sac”[Title/Abstract] OR “amnion membrane”[Title/Abstract] OR “amniotic membrane”[Title/Abstract] OR “human amniotic membrane”[Title/Abstract] OR “placental membrane”[Title/Abstract] OR “fetal membrane”[Title/Abstract] OR “amnion”[MeSH Terms]))
Web of Science (WoS)	((((((((((((TI = (“full-thickness cartilage defect”)) OR TI = (“cartilage damage”)) OR TI = (“cartilage lesions”)) OR TI = (cartilage defects)) OR TI = (“cartilage injury”)) OR TI = (“cartilage injuries”)) OR TI = (“cartilage destruction”)) OR TI = (“cartilage deterioration”)) OR TI = (“chondral lesion”)) OR TI = (“articular cartilage injury”)) OR TI = (“osteochondral defects”)) OR TI = (“cartilage diseases”)) OR (((((((((((AB = (“full-thickness cartilage defect”)) OR AB = (“cartilage damage”)) OR AB = (“cartilage lesions”)) OR AB = (“cartilage defects”)) OR AB = (“cartilage injury”)) OR AB = (“cartilage injuries”)) OR AB = (“cartilage destruction”)) OR AB = (“cartilage deterioration”)) OR AB = (“chondral lesion”)) OR AB = (“articular cartilage injury”)) OR AB = (“osteochondral defects”)) OR AB = (“cartilage diseases”) AND (((((((TI = (“human amnion”)) OR TI = (“amniotic sac”)) OR TI = (“amnion membrane”)) OR TI = (“amniotic membrane”)) OR TI = (“human amniotic membrane”)) OR TI = (“placental membrane”)) OR TI = (“fetal membrane”)) OR TI = (amnion) OR (((((((AB = (“human amnion”)) OR AB = (“amniotic sac”)) OR AB = (“amnion membrane”)) OR AB = (“amniotic membrane”)) OR AB = (“human amniotic membrane”)) OR AB = (“placental membrane”)) OR AB = (“fetal membrane”)) OR AB = (amnion)
Scopus	(TITLE-ABS (“full-thickness cartilage defect”) OR TITLE-ABS (“cartilage damage”) OR TITLE-ABS (“cartilage lesions”) OR TITLE-ABS (“cartilage defects”) OR TITLE-ABS (“cartilage injury”) OR TITLE-ABS (“cartilage injuries”) OR TITLE-ABS (“cartilage destruction”) OR TITLE-ABS (“cartilage deterioration”) OR TITLE-ABS (“chondral lesion”) OR TITLE-ABS (“articular cartilage injury”) OR TITLE-ABS (“osteochondral defects”) OR KEY (“cartilage diseases”)) AND (TITLE-ABS (“human amnion”) OR TITLE-ABS (“amniotic sac”) OR TITLE-ABS (“amnion membrane”) OR TITLE-ABS (“amniotic membrane”) OR TITLE-ABS (“human amniotic membrane”) OR TITLE-ABS (“placental membrane”) OR TITLE-ABS (“fetal membrane”) OR TITLE-ABS-KEY (“amnion”))

* All searches were conducted without the Refine/Filter settings.

## Appendix B

Table A2 shows all 20 articles included for the screening process and the decision made by both reviewers. The percentage agreement reached 85% between two reviewers.

**Table A2:** Articles included for initial title and abstract screening.

Reference	Title	Reviewers		Result Before Discussion
		Reviewer 1	Reviewer 2	
[59]	hAMSC sheet promotes repair of rabbit osteochondral defects	−1	−1	Agreement to exclude
[38]	Amniotic membrane-derived stem cells help repair osteochondral defect in a weight-bearing area in rabbits	−1	−1	Agreement to exclude
[60]	Human amniotic mesenchymal stem cell sheets encapsulating cartilage particles facilitate repair of rabbit osteochondral defects	−1	−1	Agreement to exclude
[61]	Human amniotic mesenchymal cells differentiate into chondrocytes	−1	−1	Agreement to exclude
[62]	Assessment of the in vivo biofunctionality of a biomimetic hybrid scaffold for osteochondral tissue regeneration	−1	−1	Agreement to exclude
[39]	Hypothermically stored amniotic membrane for the treatment of cartilage lesions: a single-arm prospective study with 2-year follow-up	1	1	Agreement to include
[63]	Immunological and differentiation properties of amniotic cells are retained after immobilization in pectin gel	−1	−1	Agreement to exclude
[64]	The combination of decellularized cartilage and amniotic membrane matrix enhances the production of extracellular matrix elements in human chondrocytes	1	1	Agreement to include
[36]	Osteochondral regeneration in rabbit using xenograft decellularized ECM in combination with different biological products; platelet-rich fibrin, amniotic membrane extract, and mesenchymal stromal cells	1	1	Agreement to include
[65]	Investigating the potential of human placenta-derived extracellular matrix sponges coupled with amniotic membrane-derived stem cells for osteochondral tissue engineering	−1	−1	Agreement to exclude
[66]	The influence of human amniotic fluid on the potential of rabbit ear perichondrial flaps to form cartilage tissue	−1	−1	Agreement to exclude
[67]	Isolation and characterization of human amniotic mesenchymal stem cells and their chondrogenic differentiation	−1	−1	Agreement to exclude
[68]	A human amnion-derived extracellular matrix-coated cell-free scaffold for cartilage repair: in vitro and in vivo studies	1	1	Agreement to include
[21]	Human amniotic mesenchymal stromal cells as favorable source for cartilage repair	0	−1	Conflict
[30]	Study of human acellular amniotic membrane loading bone marrow mesenchymal stem cells in repair of articular cartilage defect in rabbits	1	−1	Conflict
[35]	Human acellular amniotic membrane scaffolds encapsulating juvenile cartilage fragments accelerate the repair of rabbit osteochondral defects	1	1	Agreement to include
[28]	Human amniotic membrane as a delivery matrix for articular cartilage repair	1	1	Agreement to include
[37]	Effect of amniotic membrane/collagen scaffolds on laryngeal cartilage repair	1	1	Agreement to include
[31]	Amniotic membrane transplant for articular cartilage repair: an experimental study in sheep	1	1	Agreement to include
[29]	Effect of amniotic membrane/collagen-based scaffolds on the chondrogenic differentiation of adipose-derived stem cells and cartilage repair	1	−1	Conflict
	Percent agreement (number of agreements/total articles screened) × 100%			85%

1: Include the article. −1: Exclude the article.

## Appendix C

Table A3 lists the studies excluded from the review and the specific reasons for their exclusion.

**Table A3:** Excluded studies and the reason for exclusion for each study.

Reference	Title	Reason for Exclusion
[66]	The influence of human amniotic fluid on the potential of rabbit ear perichondrial flaps to form cartilage tissue	Use human amniotic MSCs on other scaffolds for treatment, not utilizing amnion.
[61]	Human amniotic mesenchymal cells differentiate into chondrocytes	Use human amniotic MSCs on other scaffolds for treatment, not utilizing amnion.
[67]	Isolation and characterization of human amniotic mesenchymal stem cells and their chondrogenic differentiation	Use human amniotic MSCs on other scaffolds for treatment, not utilizing amnion.
[68]	A human amnion-derived extracellular matrix-coated cell-free scaffold for cartilage repair: in vitro and in vivo studies	Use human amniotic MSCs as the scaffold materials instead of amnion itself.
[65]	Investigating the potential of human placenta-derived extracellular matrix sponges coupled with amniotic membrane-derived stem cells for osteochondral tissue engineering	Using whole decellularized placenta as the scaffold instead of using just only amnion.
[63]	Immunological and differentiation properties of amniotic cells are retained after immobilization in pectin gel	Use human amniotic MSCs on other scaffolds for treatment, not utilizing amnion.
[62]	Assessment of the in vivo biofunctionality of a biomimetic hybrid scaffold for osteochondral tissue regeneration	Use human amniotic MSCs on other scaffolds for treatment, not utilizing amnion.
[60]	Human amniotic mesenchymal stem cell sheets encapsulating cartilage particles facilitate repair of rabbit osteochondral defects	Use human amniotic MSCs as the scaffold materials instead of amnion itself.
[59]	hAMSC sheet promotes repair of rabbit osteochondral defects	Use human amniotic MSCs as the scaffold materials instead of amnion itself.
[64]	The combination of decellularized cartilage and amniotic membrane matrix enhances the production of extracellular matrix elements in human chondrocytes	Amnion is used as an extract added into the medium for cell culture instead of in the form of scaffold.

## Appendix D

Table A4 presents the risk of bias assessment for preclinical studies based on the OHAT tool.

**Table A4:** Risk of bias assessment for in vitro and in vivo studies according to the OHAT (Office of Health and Translation) tool. (Reference: [69]).

Reference	Q1	Q2	Q3	Q4	Q5	Q6	Q7	Tier
In vitro study								
Boo et al. [23]	NR	NA	+	NR	++	NR	++	Tier 2
Krishnamurithy et al. [24]	NR	NA	+	NR	++	NR	++	Tier 2
Tan et al. [25]	NR	NA	+	NR	++	NR	++	Tier 2
Lindenmair et al. [26]	NR	NA	+	NR	++	NR	++	Tier 2
Naseer et al. [27]	NR	NA	+	NR	++	NR	++	Tier 2
Ex vivo study								
Muinos-Lopez et al. [21]	NR	NR	+	NR	++	NR	++	Tier 2
Both in vitro and ex vivo study								
Díaz-Prado et al. [22]-in vitro	NR	NA	+	NR	−	NR	++	Tier 3
Díaz-Prado et al. [22]-ex vivo	NR	NR	+	NR	−	NR	++	Tier 3
Both in vitro and in vivo study								
Jin et al. [28]-in vitro	NR	NA	+	NR	++	NR	++	Tier 2
Jin et al. [28]-in vivo	NR	NR	NR	NR	++	NR	++	Tier 3
Cao et al. [29]-in vitro	NR	NA	NR	NR	NR	NR	++	Tier 3
Cao et al. [29]-in vivo	NR	NR	NR	NR	NR	NR	++	Tier 3
In vivo study								
Liu et al. [30]	++	NR	NR	NR	+	NR	+	Tier 3
Garcia et al. [31]	++	NR	NR	NR	NR	NR	−	Tier 3
Tabet et al. [32]	NR	NR	NR	NR	++	++	++	Tier 3
Tabet et al. [33]	NR	NR	NR	NR	++	++	++	Tier 3
Turgut et al. [34]	++	NR	+	NR	++	NR	++	Tier 3
Jun et al. [35]	++	NR	NR	NR	+	++	++	Tier 2
Rastegar Adib et al. [36]	NR	NR	++	NR	++	NR	++	Tier 3
Iravani et al. [37]	++	NR	+	NR	NR	NR	−−	Tier 3
Zhang et al. [38]	++	NR	NR	NR	+	NR	++	Tier 3

Q1: Was the allocation to the experimental groups randomized? Q2: Was the allocation of the study group adequately concealed? (NA for in vitro studies). Q3: Were all experimental conditions kept identical across all experimental groups? Q4: Was the researcher blinded during the conduct of the experiment? Q5: Was there any data attrition or exclusion from the final analysis without explanation? Q6: Was the assessor blinded during outcome measurement? Q7: Were all measured outcomes reported? Acronym: NR: Not reported; NA: Not applicable. ++: Definitely low risk of bias; +: Probably low risk of bias; −: Probably high risk of bias; −−: definitely high risk of bias.

## Appendix E

Table A5 presents the risk of bias assessment for clinical study [39] based on the MINORS tool.

**Table A5:** Risk of bias assessment for the non-randomized clinical study according to the MINORS (Methodological Index for Non-Randomized Studies) tool. (Reference: [70,71]).

Criteria [70,71]	Scoring (0//1/2)
Clearly stated aim	2
Inclusion of consecutive patients	2
Prospective data collection	2
Endpoints appropriate to study aim	1
Unbiased assessment of study endpoint	1
Follow-up period appropriate to study aim	2
<5% lost to follow-up	2
Prospective calculation of study size	0
Total Score:	12/16
